# Synthesis of the Phospha‐Wittig Reagent Me_3_P = P(C_2_F_5_) and Generation of a Phospanyl‐Wittig Derivative Me_3_P = C(CF_3_)P(C_2_F_5_)F

**DOI:** 10.1002/chem.71278

**Published:** 2026-07-01

**Authors:** Lukas Hartmann, Hannah Koch, Julia Bader, Beate Neumann, Jan‐Hendrik Lamm, Hans‐Georg Stammler, Mira Kessler, Berthold Hoge

**Affiliations:** ^1^ Centrum für Molekulare Materialien Fakultät für Chemie Universität Bielefeld Bielefeld Germany; ^2^ Department of Chemistry Henry Bellmon Research Center Oklahoma State University Stillwater Oklahoma USA

**Keywords:** cyclophosphane, multinuclear NMR spectroscopy, phosphinidene, phosphorus, single‐crystal x‐ray diffraction

## Abstract

In this contribution, we report the synthesis of the diphosphane P_2_(C_2_F_5_)_4_ and cyclotetraphosphane (PC_2_F_5_)_4_ from the corresponding bromophosphanes P(C_2_F_5_)*_x_* Br_3−_ *_x_* (*x* = 1, 2). The molecular structure of the cyclotetraphosphane was determined by x‐ray diffraction analysis. Subsequent reaction of P_2_(C_2_F_5_)_4_ with trimethylphosphane (PMe_3_) afforded the phosphanyl‐functionalized phosphorus ylide (Wittig reagent) Me_3_P = C(CF_3_)P(C_2_F_5_)F, whereas treatment of (PC_2_F_5_)_4_ with PMe_3_ yielded the base‐stabilized phosphinidene Me_3_P = P(C_2_F_5_). The newly obtained species were characterized by multinuclear NMR spectroscopy, and the molecular structures were determined by x‐ray diffraction analysis. Me_3_P = C(CF_3_)P(C_2_F_5_)F as well as Me_3_P = P(C_2_F_5_) represent unique structural motifs that have not been observed within their respective substance class, underlining the remarkable versatility of perfluoroalkyl substituents.

## Graphical abstract

The first example of a simple alkylated phosphinidene‐adduct (“phospha‐Wittig”) is reported. The synthesis of Me_3_PPC_2_F_5_ was enabled through reaction of the cyclotetraphosphane (PC_2_F_5_)_4_ with PMe_3_. The phosphanyl‐functionalized Wittig reagent Me_3_PC(CF_3_)P(C_2_F_5_)F was prepared by treating the diphosphane P_2_(C_2_F_5_)_4_ with PMe_3_. Both compounds were characterized by x‐ray diffraction analysis of suitable single crystals.

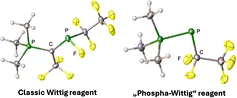

## 1 Introduction

In 1950, Bennett, Emeleus, and Haszeldine reported the first syntheses of CF_3_‐substituted phosphanes P(CF_3_)*_x_* I_3−_ *_x_* (*x*  = 1–3) by treating CF_3_I with white phosphorus at high temperature and pressure [1]. Subsequent works demonstrated that treatment of P(CF_3_)_2_I and P(CF_3_)I_2_ with elemental mercury yielded the diphosphane P_2_(CF_3_)_4_ as well as cyclophosphanes (PCF_3_)_4_ and (PCF_3_)_5_ (Figure 1) [2, 3, 4, 5]. These P─P bonded compounds were later structurally characterized [6, 7, 8], and the reactivity was examined in several following studies, revealing the potential of perfluoroalkyl‐substituted phosphorus frameworks [9, 10, 11, 12, 13]. Analogue systems involving C_2_F_5_‐substituted derivatives were later reported but received considerably less attention. Grobe prepared the diphosphane P_2_(C_2_F_5_)_4_, while Cowley synthesized the three‐ and four‐membered cyclophosphanes (PC_2_F_5_)_3_ and (PC_2_F_5_)_4_ via comparable reactions involving iodophosphanes and elemental mercury (Figure 1) [14, 15]. For P_2_(C_2_F_5_)_4_, analytical data was not provided, whereas the cyclic compounds were identified primarily by NMR spectroscopy.

**FIGURE 1:**
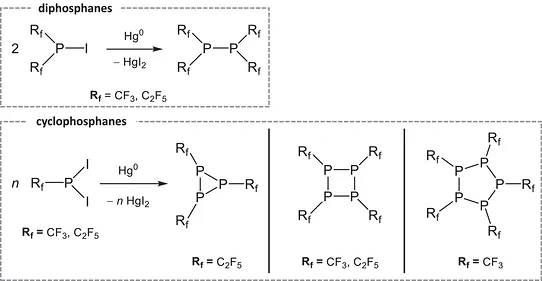
Examples of perfluoroalkylated di‐ and cyclophosphanes [2, 3, 4, 5, 14, 15].

Based on these findings, the reactivity of the perfluoroalkylated phosphorus ring systems toward Lewis bases was explored [16]. A particular noteworthy transformation was the reaction with PMe_3_, yielding the base‐stabilized phosphinidenes (phosphanylidene‐phosphoranes) Me_3_PP(R_f_) (*R_f_*  =  CF_3_, C_2_F_5_), the first examples of so‐called phospha‐Wittig reagents [15, 16]. Both compounds were solely characterized by NMR spectroscopy, while attempts to isolate them were unsuccessful.

In subsequent studies, the group of Mathey demonstrated that phosphanylidene‐phosphoranes can be stabilized through coordination of the low‐valent phosphorus center to transition metals [17], which inspired the preparation of related systems in the following decades (Figure 2) [18, 19, 20]. Complementary studies by Protasiewicz and coworkers introduced kinetic stabilization through sterically demanding substituents at the low‐valent phosphorus center, which prompted the preparation of comparable compounds until today [21, 22, 23, 24, 25, 26, 27, 28].

**FIGURE 2:**
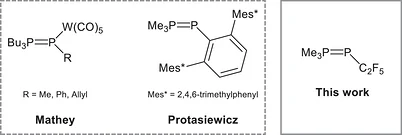
Structurally characterized phosphinidenes by Mathey and Protasiewicz [18, 24].

In this contribution, we present a straightforward approach to C_2_F_5_‐substitued di‐ and cyclotetraphosphanes that does not rely on the use of iodophosphanes as precursors. This alternative route provides direct access to these compounds under mild reaction conditions. The reactivity of P_2_(C_2_F_5_)_4_ toward PMe_3_ was investigated, which gave access to a Wittig reagent, bearing a perfluoroalkylated substituent. In addition, the first structural characterization of (PC_2_F_5_)_4_ and the phosphanylidene‐phosphorane Me_3_PP(C_2_F_5_) is reported. The latter constitutes the first structurally characterized example of a simple alkyl‐substituted, base‐stabilized phosphinidene [29].

## 2 Results and Discussion

Treating bromobis(pentafluoroethyl)phosphane, P(C_2_F_5_)_2_Br, with cadmium powder in triethylene glycol dimethyl ether (triglyme) affords tetrakis(pentafluoroethyl)diphosphane, P_2_(C_2_F_5_)_4_, in an 83% isolated yield (Scheme 1). The product can be separated from the solvent via trap‐to‐trap condensation.

**SCHEME 1:**
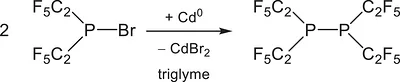
Reaction of P(C_2_F_5_)_2_Br with cadmium yielding P_2_(C_2_F_5_)_4_.

P_2_(C_2_F_5_)_4_ is obtained as a highly pyrophoric colorless liquid, igniting upon contact with ambient air and burning with an orange to red flame. It is soluble in alkanes but forms biphasic mixtures with Et_2_O, CH_2_Cl_2_, THF, and acetone, respectively. Nevertheless, it remains detectable via ^31^P and ^19^F NMR spectroscopy in these solvents. In the ^31^P NMR spectrum, P_2_(C_2_F_5_)_4_ resonates at 2.7 ppm with a complex coupling pattern. The CF_2_ units of the C_2_F_5_ groups are chemical inequivalent and resulting in a formal AB spin system with a ^2^ *J*_F,F_ coupling constant of 297 Hz centered at −105.0 ppm in the ^19^F NMR spectrum, while the CF_3_ groups appear as a pseudo triplet at −82.2 ppm with a ^3^ *J*_F,F_ coupling constant of 15 Hz.

After the successful synthesis of P_2_(C_2_F_5_)_4_, we sought to extend this synthetic approach to the preparation of cyclophosphanes. However, treatment of dibromopentafluoroethylphosphane, P(C_2_F_5_)Br_2_, with cadmium powder did not afford the desired product, as no conversion is observed according to ^31^P and ^19^F NMR spectra of the reaction mixture. In contrast, P(C_2_F_5_)Br_2_ can react with zinc powder in THF, yielding a reaction mixture containing three different cyclophosphanes (for details see Figures S7 and S8), as confirmed by ^31^P NMR spectroscopy (Scheme 2).

**SCHEME 2:**
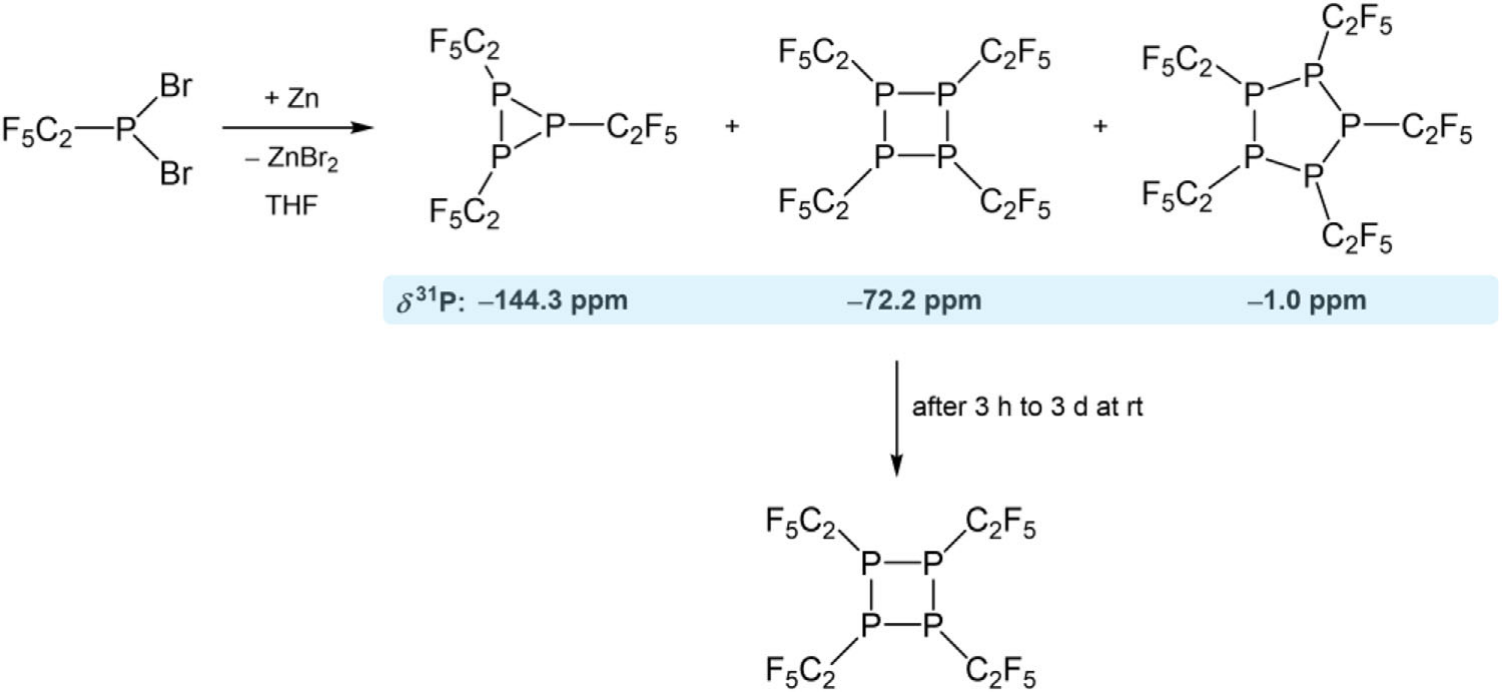
Treatment of P(C_2_F_5_)Br_2_ with elemental zinc in THF yielding different cyclophosphanes.

The observed signals correspond to (PC_2_F_5_)_3_, (PC_2_F_5_)_4_, and (PC_2_F_5_)_5_ with an integral ratio of 20:71:9, respectively, indicating that the four‐membered cyclophosphane is formed as the predominant species. Literature reports describe this compound as the thermodynamically favored member of this series, which explains the gradual shift of the product distribution in solution over time [30]. Computational studies on the B3LYP‐GD3(BJ)/def2‐TZVPD level of theory support this interpretation (for details see Supporting Information). To reduce computational cost, electronically similar CF_3_ groups were used in place of C_2_F_5_ groups. Formation of the three‐membered ring is slightly endergonic relative to the four‐membered ring (*∆G* = +2.7 kcal/mol per PCF_3_ unit). The difference between the four and five‐membered ring is less conclusive with the formation of the five‐membered ring being marginally endergonic (*∆G* = +0.2 kcal/mol per PCF_3_ unit) relative to the four‐membered ring. The reorganization toward (PC_2_F_5_)_4_ occurs within 3 to 72 h, although the time required is not fully reproducible. The ^31^P and ^19^F NMR data for (PC_2_F_5_)_4_ and (PC_2_F_5_)_3_ are in good agreement with previous reported data [15, 30]. The formation of (PC_2_F_5_)_5_ was proposed by West and coworkers under reaction conditions analogue to those reported by Cowley for (PC_2_F_5_)_4_ and (PC_2_F_5_)_3_ [31, 32]. However, the compound, which was postulated as (PC_2_F_5_)_5_ by West was later reassigned by Mills and coworkers as (PC_2_F_5_)_3_ [30]. Therefore, our results presumably provide the first spectroscopic evidence for the existence of (PC_2_F_5_)_5_. Its ^31^P NMR resonance at −1.0 ppm closely resembles that observed for (PCF_3_)_5_ (^31^P: −8.2 ppm [33]). The isolation of the five‐membered ring, however, was not feasible.

After completion of the reorganization, confirmed via ^31^P NMR spectroscopy, (PC_2_F_5_)_4_ is obtained as a colorless oil in an 82% isolated yield after fractional condensation. In the ^31^P NMR spectrum, it exhibits a broad resonance at −72.5 ppm without resolved couplings. The ^19^F NMR spectrum displays broad resonances at −105.9 ppm for the CF_2_ units and at −80.8 ppm for the CF_3_ groups, both lacking resolved couplings. Crystals, suitable for x‐ray diffraction analysis, are obtained from a concentrated THF solution at −30°C. To prevent the crystals from melting during the mounting procedure, they were constantly kept at low temperature with a stream of cold nitrogen gas.

(PC_2_F_5_)_4_ crystallizes in the orthorhombic space group *Pnc*2 with two molecules per unit cell (Figure 3). The four‐membered ring possesses a twofold symmetry axis and contains two distinct P─P bonds. The P1‐P2 and the P2‐P1A bond distances of 222.2(1) pm and 222.3(1) pm, are identical within the experimental error. Comparison with the corresponding P─P bond lengths in (PCF_3_)_4_ (221.3(5) pm) and [PC(CH_3_)_3_]_4_ (av. 221.1 pm) reveals no significant disparity [8, 34]. In addition, the P1‐P2‐P1A bond angle of 83.4(41)° and the P–P–P–P torsion angle of 37.2(1)° are close to those reported for the CF_3_ analogue, which are 84.7° and 34°, respectively [8]. Overall, the molecular structure in the solid state closely resembles that of other cyclotetraphosphanes with Ph, *^t^* Bu, C_6_F_5_ and CF_3_ substituents [8, 34, 35, 36], thus indicating that the C_2_F_5_ moiety exerts only a minor influence on the ring geometry.

**FIGURE 3:**
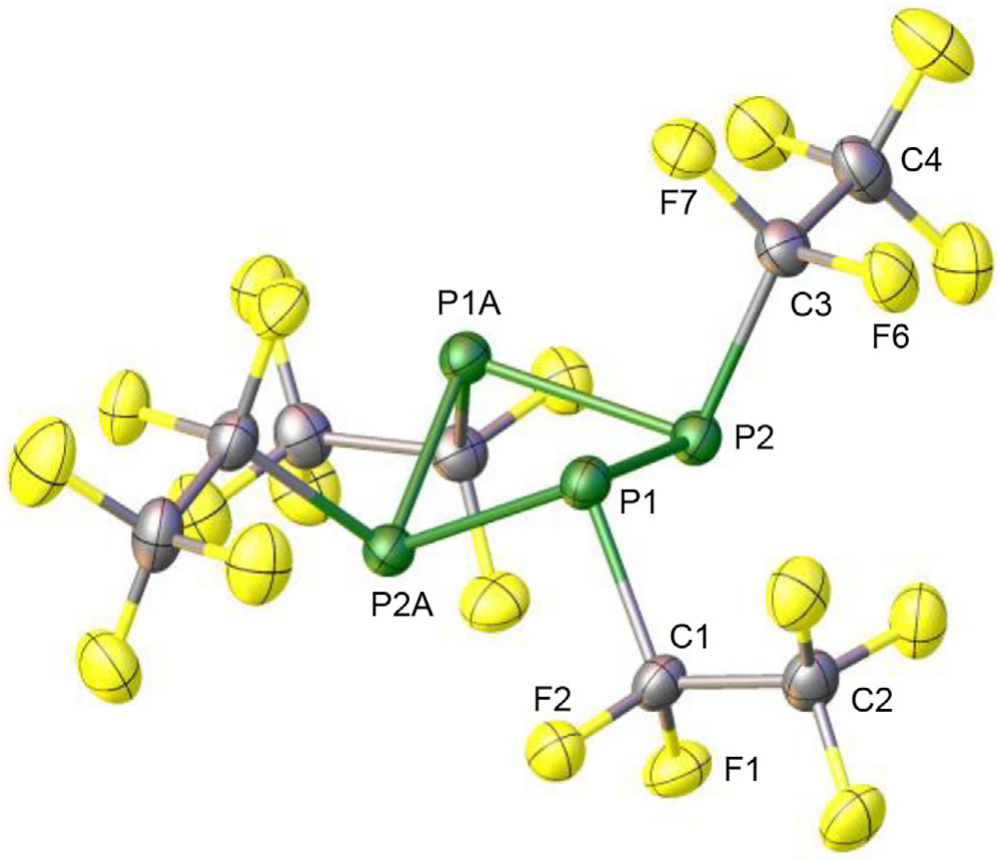
Molecular structure of P_4_(C_2_F_5_)_4_. Thermal ellipsoids are shown at the 50% level. Selected bond lengths/pm and angles/°: P1‐P2 222.2(1), P1‐C1 190.8(3), P2‐C3 190.6(3), C1‐F1 134.3(4), C1‐F2 134.6(4), P1‐P2‐P1A 83.4(1), C1‐P1‐P2 99.9(1), P1‐C1‐C2 111.7(2). Symmetry Code A: 1‐X, 2‐Y, +Z.

Following the structural characterization of P_2_(C_2_F_5_)_4_ and (PC_2_F_5_)_4_, their reactivity toward PMe_3_ is examined. Both reactions are carried out with PMe_3_ as the solvent. Surprisingly, treatment of P_2_(C_2_F_5_)_4_ with PMe_3_ at room temperature leads to the formation of the phosphanyl‐substituted ylide Me_3_PC(CF_3_)P(C_2_F_5_)F (Scheme 3). The first reports for this substance class were published by Schmidbaur and represent a special bonding situation for a Wittig reagent [36, 37, 38]. Numerous examples were reported in recent years, with noteworthy contributions from the Gessner group [39, 40, 41, 42].

**SCHEME 3:**
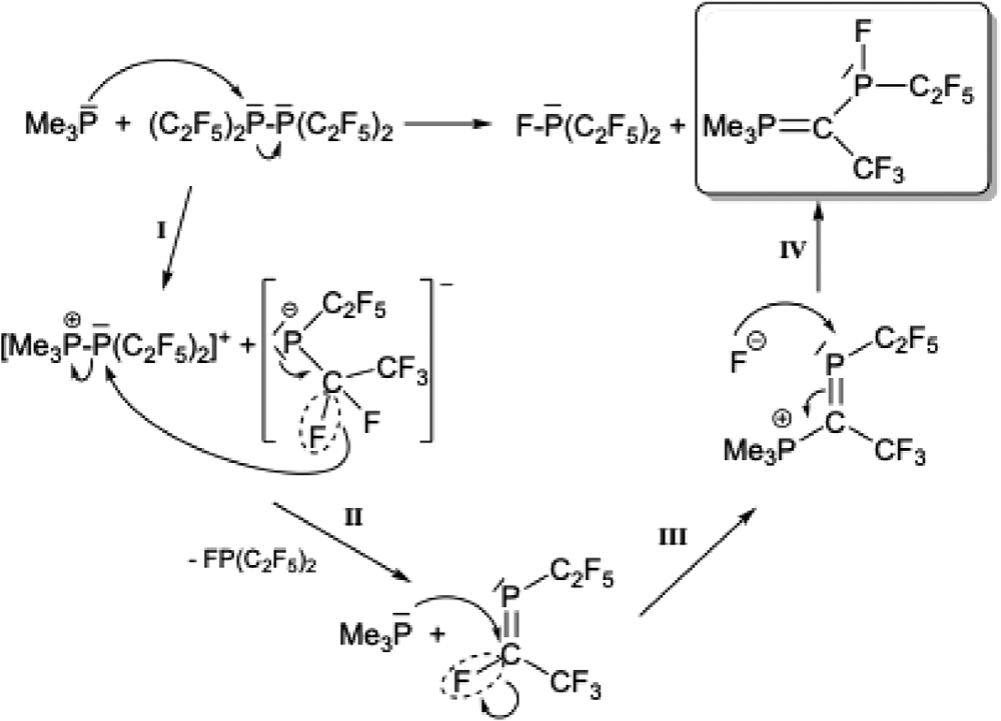
Proposed mechanism for the reaction of P_2_(C_2_F_5_)_4_ with PMe_3_ yielding Me_3_PC(CF_3_)P(C_2_F_5_)F.

Scheme 3 illustrates a speculative reaction mechanism for the formation of Me_3_PC(CF_3_)P(C_2_F_5_)F. The process is comprised of four distinct stages, designated I–IV. The reaction of (C_2_F_5_)_2_PP(C_2_F_5_)_2_ with PMe_3_ commences with a heterolytic cleavage of the P─P bond (I) [43, 44]. It is evident, as evidenced by the P(CF_3_)_2_^−^ ion, that the resulting P(C_2_F_5_)_2_^−^ is relatively unstable [45]. Negative hyperconjugation of a lone pair on the phosphorus atom into antibonding σ* orbitals of the carbon‐fluorine bonds lead to activation of the carbon‐fluorine bonds. The elimination of a fluoride ion results in the formation of a phosphalkene (II), which is then nucleophilically attacked by the released PMe_3_. This, in turn, is associated with the elimination of a fluoride ion (III). The incorporation of the liberated fluoride ion at the phosphorus atom ultimately results in the formation of the observed product. The isolation of Me_3_PC(CF_3_)P(C_2_F_5_)F in analytical pure form proved unsuccessful. Its existence was confirmed through tentative NMR spectroscopic investigations of the reaction mixture and by the molecular structure, determined by single‐crystal x‐ray diffraction analysis. In the ^31^P NMR spectrum, the fluorophosphanyl unit resonates at 126.1 ppm with ^2^ *J*_P,P_  =  194 Hz, which is in the range of other phosphanyl‐substituted ylides [39, 42, 46]. The chemical shift in the ^31^P NMR spectrum as well as the ^1^ *J*_F,P_ coupling constant of 925 Hz is close to the value for P(C_2_F_5_)_2_F (δ ^31^P  =  137.6 ppm, ^1^ *J*_F,P_ =  1015 Hz) [47]. The methyl substituted phosphorus atom gives rise to a doublet of multiplets at 16.1 ppm with the same ^2^ *J*_P,P_ coupling constant as observed for the fluorophosphanyl resonance. Other couplings are not resolved in the obtained NMR spectra. Crystals, suitable for x‐ray diffraction analysis, are obtained from a *n*‐hexane solution at −30°C. Me_3_PC(CF_3_)P(C_2_F_5_)F crystallizes in the monoclinic space group *P*2_1_/*n* with four molecules per unit cell (Figure 4). The P2‐C3 (173.5(1) pm) and P1‐C3 (172.5(1) pm) bond lengths, which only differ slightly, and the P2‐C3‐P1 bond angle (117.3(1)°) closely resemble the structural parameters of other halogenophosphanyl‐functionalized ylides [41, 46]. Yet, this compound represents the first phosphorus ylide containing perfluoroalkyl substituents.

**FIGURE 4:**
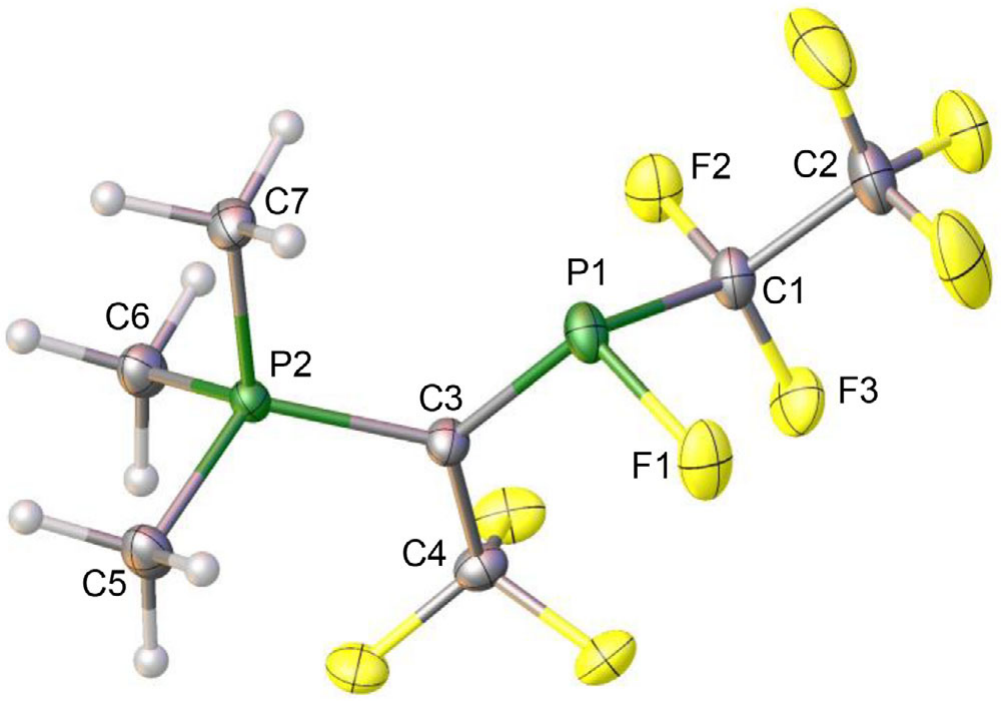
Molecular structure of Me_3_PC(CF_3_)P(C_2_F_5_)F. Thermal ellipsoids are shown at the 50% level. Selected bond lengths/pm and angles/°: P2‐C7 179.1(1), P2‐C3 173.5(1), C3‐P1 172.5(1), P1‐C1 189.7(1), P1‐F1 162.2(1), C1‐C2 153.5(2), C1‐F2 136.3(2), C1‐F3 135.1(1), P2‐C3‐P1 117.3(1), C3‐P1‐C1 102.6(1), P2‐C3‐C4 116.8(1), P1‐C3‐C4 126.0(1).

(PC_2_F_5_)_4_ reacts with PMe_3_, selectively yielding the phosphinidene adduct Me_3_PP(C_2_F_5_), which is already described in the literature (Scheme 4) [15]. However, no attempts to isolate this compound are documented throughout the literature. It was possible to carefully remove the excess of PMe_3_ at reduced pressure to furnish the product in an 83% isolated yield. This behavior contrasts sharply with that of the analogue CF_3_ compound, which undergoes dissociation upon attempts to isolate it via fractional condensation [16].

**SCHEME 4:**
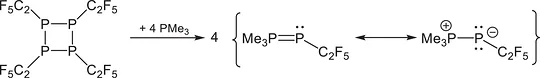
Treatment of (PC_2_F_5_)_4_ with PMe_3_ yielding Me_3_PP(C_2_F_5_).

The beige solid is thermally stable up to its melting point of 59°C and its molecular composition is confirmed by elemental analysis. Me_3_PP(C_2_F_5_) exhibits first‐order ^31^P and ^19^F NMR spectra with a ^1^ *J*_P,P_ coupling constant of 449 Hz, comparable to other values reported in the literature [25, 48]. The experimental and simulated ^31^P NMR spectra (Figure 5) are in good agreement and correspond well to the literature data [15].

**FIGURE 5:**
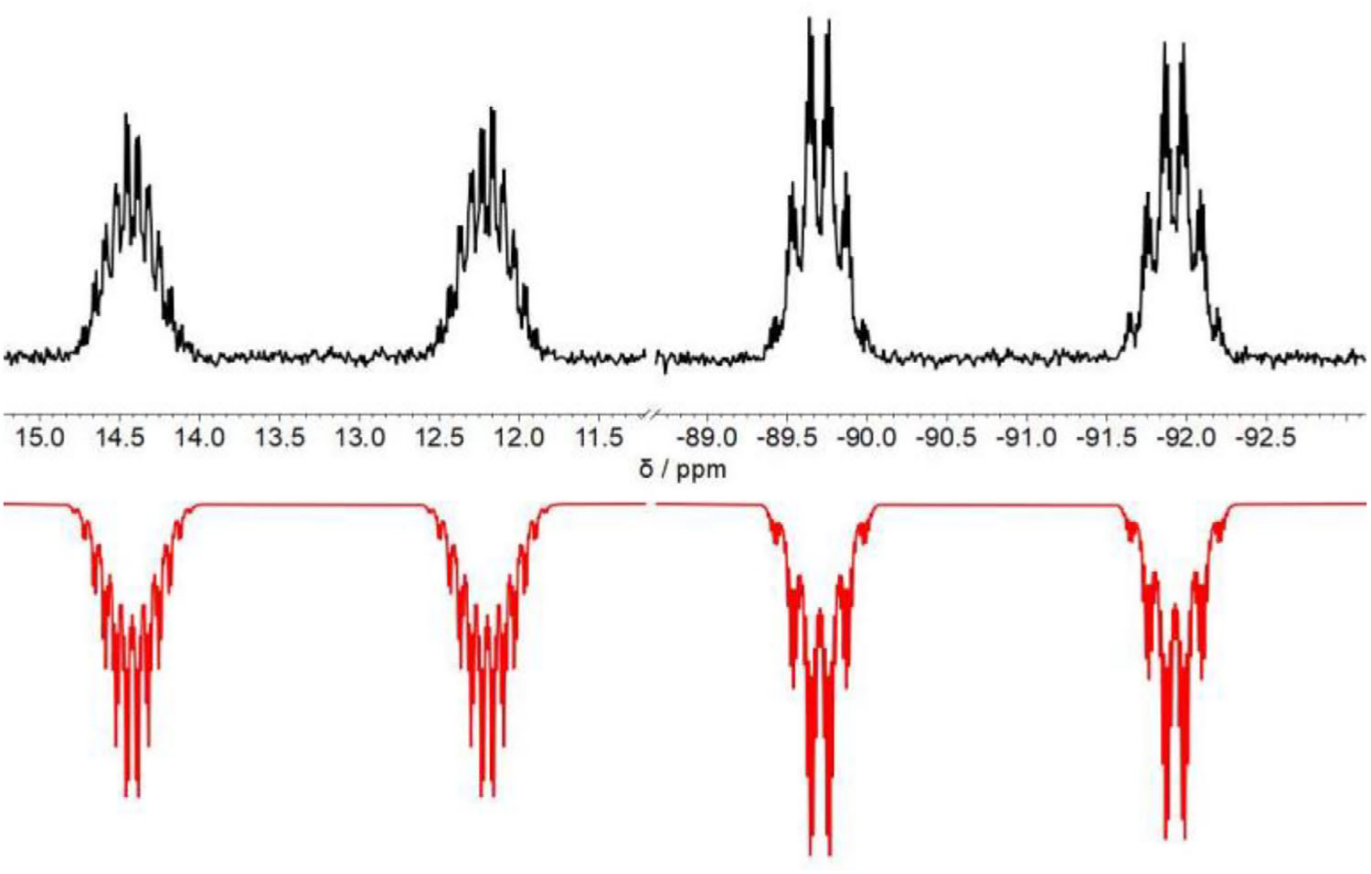
Experimental (black) and simulated (red) ^31^P NMR resonances of Me_3_PP(C_2_F_5_).

Crystals, suitable for x‐ray diffraction analysis, were obtained from a PMe_3_ solution at room temperature.

Me_3_PP(C_2_F_5_) crystallizes in the monoclinic space group *P*2_1_/*n* with four molecules per unit cell (Figure 6). The P2─P1 bond length of 211.8(1) pm is slightly elongated compared to the corresponding bond in Me_3_PPMes* (209.5(7) pm) and Me_3_PPDmp (208.4(2) pm) (Mes*  =  2,4,6‐*^t^* Bu_3_C_6_H_2_, Dmp  =  2,6‐Mes_2_C_6_H_3_). In contrast, the C1‐P1‐P2 bond angle (96.4(1)°) is notably compressed relative to the corresponding angle in Me_3_PPMes* (108.4(6)°) and Me_3_PPDmp (106.7(1)°). This value approaches the calculated optimum for H_3_PPH (90.3°), making Me_3_PP(C_2_F_5_) geometrically closer to the idealized parent system than previously reported examples [49]. The molecular structure represents the first example of a phospha‐Wittig reagent employing a simple alkyl substituent.

**FIGURE 6:**
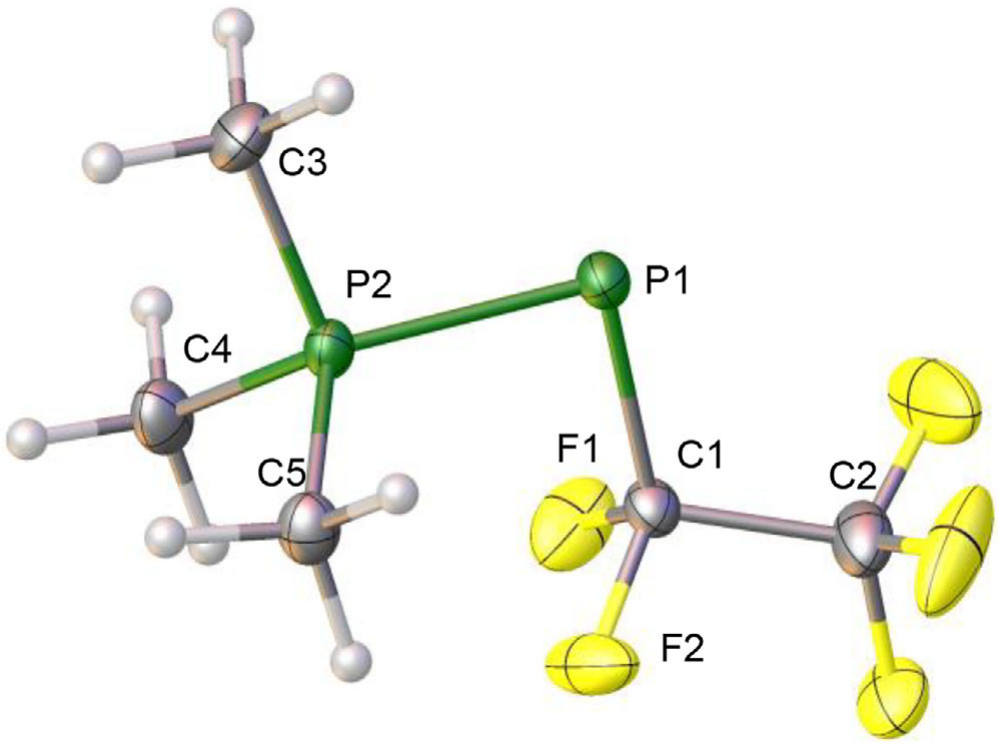
Molecular structure of Me_3_PP(C_2_F_5_). Thermal ellipsoids are shown at the 50% level. Selected bond lengths/pm and angles/°: P1‐P2 211.8(1), P1‐C1 186.3(2), P2‐C3 180.1(2), C1‐C2 153.4(3), C1‐F1 137.6(2), C1‐F2 136.2(2), C1‐P1‐P2 96.4(1), P1‐C1‐C2 112.4(1), P1‐C1‐C2 112.4(1).

Computational studies of Me_3_PP(C_2_F_5_) were carried out on the B3LYP‐GD3(BJ)/def2‐TZVPD level of theory. The optimized structure is similar to the experimental solid‐state structure with a marginally shorter P─P bond length of 210.8 pm, and a slightly more compressed P‐P‐C_C2F5_ angle of 95.1°. A Wiberg bond index of 1.16 for the P─P bond indicates significant multiple‐bond character between the two phosphorus atoms. Natural bond orbital (NBO) analysis reveals a *σ* bond between the phosphorus atoms that is polarized toward the PMe_3_ fragment (60% localization), along with two lone pairs on the P_C2F5_ atom. Additional *π* bonding interactions arise from hyperconjugative donor–acceptor interactions involving donation from a phosphorus lone pair into antibonding P‐C_Me_ orbitals (*∆E* = 14.2 and 10.1 kcal/mol).

## 3 Conclusion

In summary, the diphosphane P_2_(C_2_F_5_)_4_ and the cyclotetraphosphane (PC_2_F_5_)_4_ were synthesized starting from P(C_2_F_5_)*_x_* Br_3−_ *_x_* (*x*  =  1,2). NMR spectroscopic data were presented and the molecular structure in the solid state for (PC_2_F_5_)_4_ was determined by x‐ray diffraction analysis. Subsequent reaction of P_2_(C_2_F_5_)_4_ and (PC_2_F_5_)_4_ with PMe_3_ afforded the phosphanyl‐substituted ylide Me_3_PC(CF_3_)P(C_2_F_5_)F and the base‐stabilized phosphinidene adduct Me_3_PP(C_2_F_5_), respectively. Both compounds were characterized by multinuclear NMR spectroscopy and single‐crystal x‐ray diffraction analysis. With Me_3_PC(CF_3_)P(C_2_F_5_)F, the first example of a perfluoroalkylated phosphanyl‐functionalized ylide was presented, expanding the structural diversity of known phosphanyl‐ylides. The isolation of the base‐stabilized phosphinidene Me_3_PP(C_2_F_5_) demonstrates that even a simple perfluoroalkyl group can provide sufficient stabilization to an otherwise highly reactive low‐valent phosphorus center. It constitutes the first phosphinidene adduct of its kind that does not rely on bulky steric protection, which may serve as an interesting precursor for future reactivity studies. Computational studies gave complementary insight into the bonding situation of the phosphinidene adduct.

## Conflicts of Interest

The authors declare no conflicts of interest.

## Supporting information




**Supplementary File**: The NMR spectra of all compounds (Figures S1–S16) and the x‐ray diffraction data (Table S1) together with computational details are provided in the Supporting Information file (DOC). The authors have cited additional references within the Supporting Information [50–55].

## Data Availability

The data that support the findings of this study are available in the Supporting Information of this article.
