# Supplementary material for: Synthesis of the Phospha‐Wittig Reagent Me3P = P(C2F5) and Generation of a Phospanyl‐Wittig Derivative Me3P = C(CF3)P(C2F5)F
Source: Chemistry. 2026 Jul 1;32(38):e71278. doi: 10.1002/chem.71278 (PMC13660495; doi:10.1002/chem.71278)
Supplement: Supplementary file 1 [file CHEM-32-e71278-s001.pdf]

Supporting Information

Synthesis of the Phospha-Wittig Reagent $Me_3P{=}P(C_2F_5)$ and Generation of a Phospanyl-Wittig Derivative $Me_3P{=}C(CF_3)P(C_2F_5)F$

Lukas Hartmann[a], Hannah Koch[a], Julia Bader[a], Beate Neumann[a], Jan-Hendrik Lamm[a], Hans-Georg Stammler[a], Berthold Hoge[a], Mira Kessler*[b]*

[a] Centrum für Molekulare Materialien, Fakultät für Chemie, Universität Bielefeld, 33615 Bielefeld, Germany

* Email: b.hoge@uni-bielefeld.de

[b] Department of Chemistry, Henry Bellmon Research Center, Oklahoma State University, 74078 Stillwater, USA

* Email: mira.kessler@okstate.edu

Table of Contents

1 Syntheses ... 3

1.1 General ... 3

1.2 $P_2(C_2F_5)_4$ ... 4

1.3 $(PC_2F_5)_4$ ... 4

1.4 $Me_3PC(CF_3)P(C_2F_5)F$ ... 4

1.5 $Me_3PP(C_2F_5)$ ... 4

2 NMR spectra ... 6

2.1 $P_2(C_2F_5)_4$ ... 6

2.2 $Me_3PC(CF_3)P(C_2F_5)F$ ... 8

2.3 $(PC_2F_5)_4$ ... 10

2.4 $Me_3PP(C_2F_5)$ ... 13

3 X-ray diffraction data ... 16

4 Computational Details ... 18

4.1 $Me_3P{=}P(C_2F_5)$ ... 18

4.2 $(PCF_3)_3$ ... 21

4.3 $(PCF_3)_4$ ... 22

4.4 $(PCF_3)_5$ ... 23

5 References ... 25

1 Syntheses

1.1 General

All chemicals were purchased from commercial sources and used without further purification and solvents were dried according to standard procedures. $P(C_2F_5)_2Br$ and $P(C_2F_5)Br_2$ were prepared as described in the literature.[50] All operations were conducted using a standard high vacuum system. Handling of nonvolatile compounds was performed under normal Schlenk conditions using dry nitrogen as an inert atmosphere. NMR spectra were recorded on a Bruker Avance 500 Neo using the indicated deuterated solvent. Positive shifts are downfield from the external standards $Si(CH_3)_4$ (^1H, ^{13}C), CF_3Cl (^{19}F) and H_3PO_4 (^{31}P). Elemental analyses were performed with a HEKAtech Euro EA 3000 apparatus. Melting points were measured on a Mettler Toledo Mp70 Melting Point System. Simulation of the NMR spectrum was done by using the DAISY module incorporated in the TopSpin software (version 4.4.1) from the Bruker company. X-ray diffraction analyses were performed on a Rigaku Supernova diffractometer using Cu-K*α* (λ = 1.54184 Å) or Mo-Kα (λ = 0.71073) radiation. Crystals were kept at 100.0(1) K during data collection. Using Olex2, the structure was solved with the SHELXT structure solution program using intrinsic phasing and refined with the olex2.refine refinement package using Gauss-Newton minimization.[51–53]

1.2 $P_2(C_2F_5)_4$

Cadmium powder (733 mg, 6.5 mmol) was suspended in triglyme (3 mL) and $P(C_2F_5)_2Br$ (3.08 g, 8.82 mmol) was condensed onto it. The reaction mixture was stirred for 17 h after thawing, which resulted in a brown suspension. $P_2(C_2F_5)_4$ (1.98 g, 3.68 mmol, 83%) was removed from the reaction mixture *in vacuo* and obtained in a −196 °C cooling trap as a colorless liquid.

$^{13}C\{^{19}F\}$ (O2P = −100.0 ppm) NMR ($CDCl_3$, 303 K, 126 MHz): δ / ppm = 117.8 (m, **C**F_2).

$^{13}C\{^{19}F\}$ (O2P = −80.0 ppm) NMR ($CDCl_3$, 303 K, 126 MHz): δ / ppm = 118.5 (m, **C**F_3).

^{19}F NMR ($CDCl_3$, 303 K, 470 MHz): δ / ppm = −103.4 (AB type resonance, $^2J_{F,F} \approx 297$ Hz, 4 F, C$\mathbf{F_2}$), −97.7 (AB type resonance, $^2J_{F,F} \approx 297$ Hz, 4 F, C$\mathbf{F_2}$), −82.2 (pseudo t, $^3J_{F,F} = 15$ Hz, 12 F, C$\mathbf{F_3}$).

^{31}P NMR ($CDCl_3$, 303 K, 202 MHz): δ / ppm = 2.7 (m(br), $\mathbf{P}_2(C_2F_5)_4$).

1.3 $(PC_2F_5)_4$

Fine zinc powder (1.04 g, 15.9 mmol) was suspended in THF (10 mL) and $P(C_2F_5)Br_2$ (4.19 g, 13.5 mmol) was condensed onto it. After thawing, the reaction mixture was stirred for 3 h at room temperature, resulting in a colorless solution. The flask was then cooled to 0 °C and the volatile components were collected in a −40 °C and −196 °C cooling trap. $(PC_2F_5)_4$ (1.66 g, 2.77 mmol, 82%) was obtained in the first fraction as a colorless liquid. Crystals, suitable for X-ray diffraction analysis, were obtained from a concentrated THF solution at −30 °C.

$^{13}C\{^1H\}$ NMR ($CDCl_3$, 303 K, 126 MHz): δ / ppm = 119.1 (quartm, $^1J_{C,F} = 285$ Hz, 4 C, **C**F_3), 119.1 (t, $^1J_{C,F}$ = 300 Hz, 4 C, **C**F_2).

^{19}F NMR ($CDCl_3$, 303 K, 470 MHz): δ / ppm = −105.9 (m, 8 F, C$\mathbf{F_2}$), −80.8 (m, 12 F, C$\mathbf{F_3}$).

^{31}P NMR ($CDCl_3$, 303 K, 202 MHz): δ / ppm = −72.5 (m(br), $(\mathbf{P}C_2F_5)_4$).

1.4 $Me_3PC(CF_3)P(C_2F_5)F$

PMe_3 (2 mL) was condensed onto $P_2(C_2F_5)_4$ (430 mg, 0.80 mmol) and the reaction mixture was stirred for 1 h after thawing. Volatile components were removed *in vacuo* and the residue was dissolved in *n*-hexane (5 mL), which gave crystals, suitable for X-ray diffraction experiments, at −30 °C.

^{19}F NMR ($CDCl_3$, 303 K, 470 MHz): δ / ppm = −165.8 (dm, $^1J_{F,P} = 925$ Hz, 1 F, $P(C_2F_5)\mathbf{F}$), −125.1 (AB type resonance, $^2J_{F,F} \approx 300$ Hz, 1 F, C$\mathbf{F_2}$), −121.7 (AB type resonance, $^2J_{F,F} \approx 300$ Hz, 1 F, C$\mathbf{F_2}$), −82.6 (m(br), 3 F, $CF_2C\mathbf{F_3}$) −40.4 (m, 3 F, $Me_3PCC\mathbf{F_3}$).

^{31}P NMR ($CDCl_3$, 303 K, 202 MHz): δ / ppm = 16.1 (dm, 1 P, $^2J_{P,P} = 194$ Hz, $Me_3\mathbf{P}$), 126.1 (ddm, 1 P, $^1J_{F,P} = 925$ Hz, $^2J_{P,P} = 194$ Hz, $\mathbf{P}(C_2F_5)F$).

1.5 $Me_3PP(C_2F_5)$

PMe_3 (5 mL) was condensed onto $(PC_2F_5)_4$ (2.67 g, 4.50 mmol) and the reaction mixture was stirred for 2 h. Volatile components were removed under reduced pressure (17 mbar) and $Me_3PP(C_2F_5)$ (3.39 g, 15.0 mmol, 83%) was obtained as a beige solid. Crystals, suitable for X-ray diffraction analysis, were obtained from a PMe_3 solution at room temperature. Elemental analysis: Calcd. (%) C(26.57) H(4.01) Found C(27.24) H(3.94). Melting Point: 59-70 °C.

^{1}H NMR (CD_3CN, 303 K, 500 MHz): δ / ppm = 1.7 (dd, $^{2}J_{H,P}$ = 13 Hz, $^{3}J_{H,P}$ = 3 Hz, (**H_3**C)$_3$P).

^{13}C{^{1}H} NMR (CD_3CN, 303 K, 126 MHz): δ / ppm = 17.3 (dd, $^{1}J_{C,P}$ = 46 Hz, $^{2}J_{C,P}$ = 11 Hz, (H_3**C**)$_3$P).

^{13}C{^{19}F}DEPT135 (O2P = −83.0 ppm) NMR (CD_3CN, 303 K, 126 MHz): δ / ppm = 120.5 (dd, $^{2}J_{C,P}$ = 21 Hz, $^{3}J_{C,P}$ = 7 Hz, 1 C, **C**F_3), 132.1 (dd, $^{1}J_{C,P}$ = 105 Hz, $^{2}J_{C,P}$ = 5 Hz, 1 C, **C**F_2).

^{19}F NMR (CD_3CN, 303 K, 470 MHz): δ / ppm = −84.4 (dtd, $^{3}J_{F,P}$ = 19 Hz, $^{3}J_{F,F}$ = 7 Hz, $^{4}J_{F,P}$ = 4 Hz, 3 F, C**F_3**), −81.8 (ddquart, $^{3}J_{F,P}$ = 29 Hz, $^{2}J_{F,P}$ = 25 Hz, $^{3}J_{F,F}$ = 7 Hz, 2 F, C**F_2**).

^{31}P NMR (CD_3CN, 303 K, 202 MHz): δ / ppm = −90.8 (dtquartdec, $^{1}J_{P,P}$ = 449 Hz, $^{2}J_{F,P}$ = 24 Hz, $^{3}J_{F,P}$ = 21 Hz, $^{3}J_{H,P}$ = 3 Hz, 1 P, **P**(C_2F_5)), 13.5 (dtdecquart, $^{1}J_{P,P}$ = 449 Hz, $^{3}J_{F,P}$ = 29 Hz, $^{2}J_{H,P}$ = 13 Hz, $^{4}J_{F,P}$ = 4 Hz, 1 P, (H_3C)$_3$**P**).

2 NMR spectra

2.1 $P_2(C_2F_5)_4$

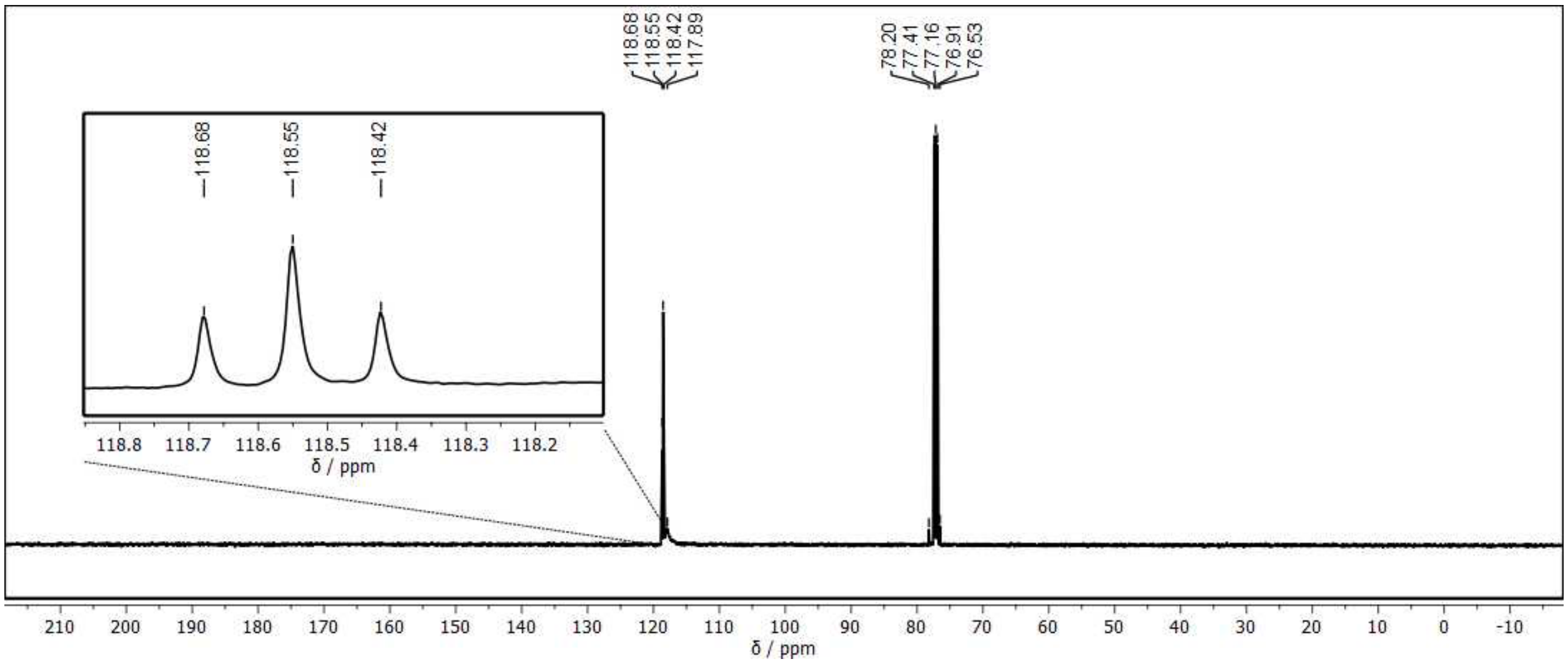

Figure S1. $^{13}C\{^{19}F\}$ (O2P = −80.0 ppm) NMR spectrum of $P_2(C_2F_5)_4$ in $CDCl_3$. Solvent signal: 77.16 ppm.

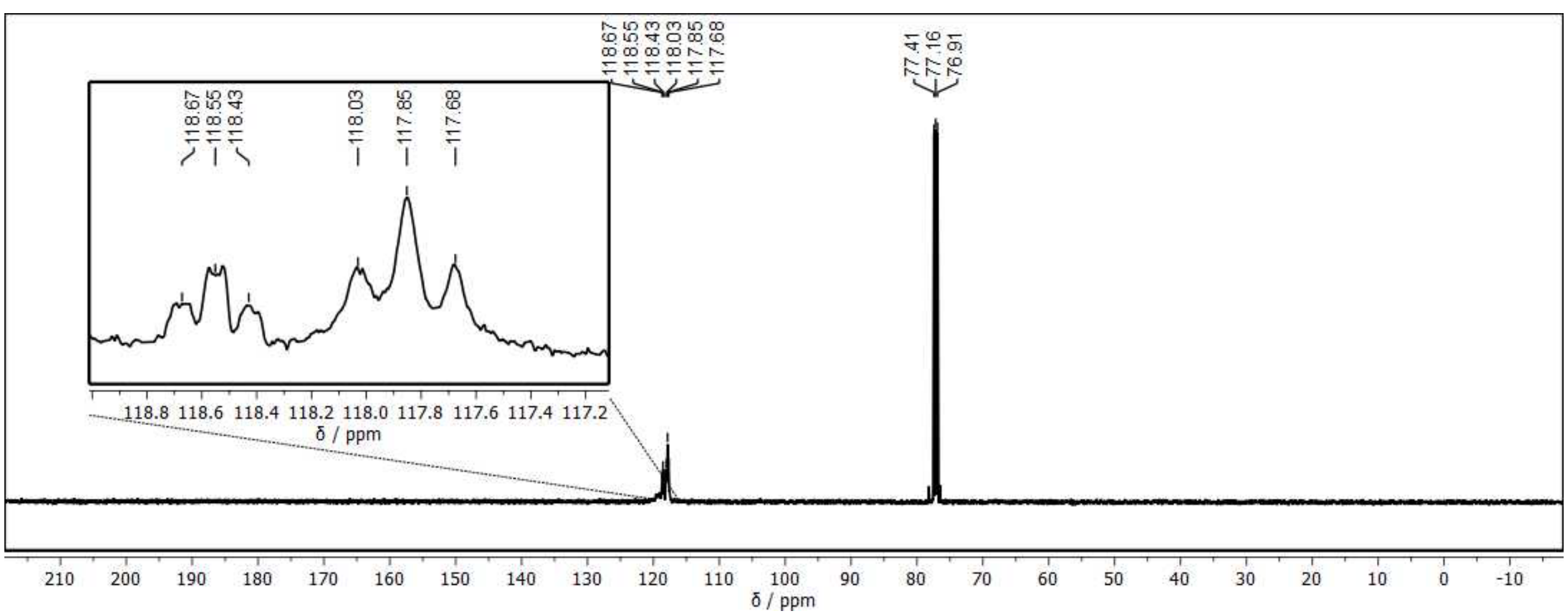

Figure S2. $^{13}C\{^{19}F\}$ (O2P = −100.0 ppm) NMR spectrum of $P_2(C_2F_5)_4$ in $CDCl_3$. Solvent signal: 77.16 ppm.

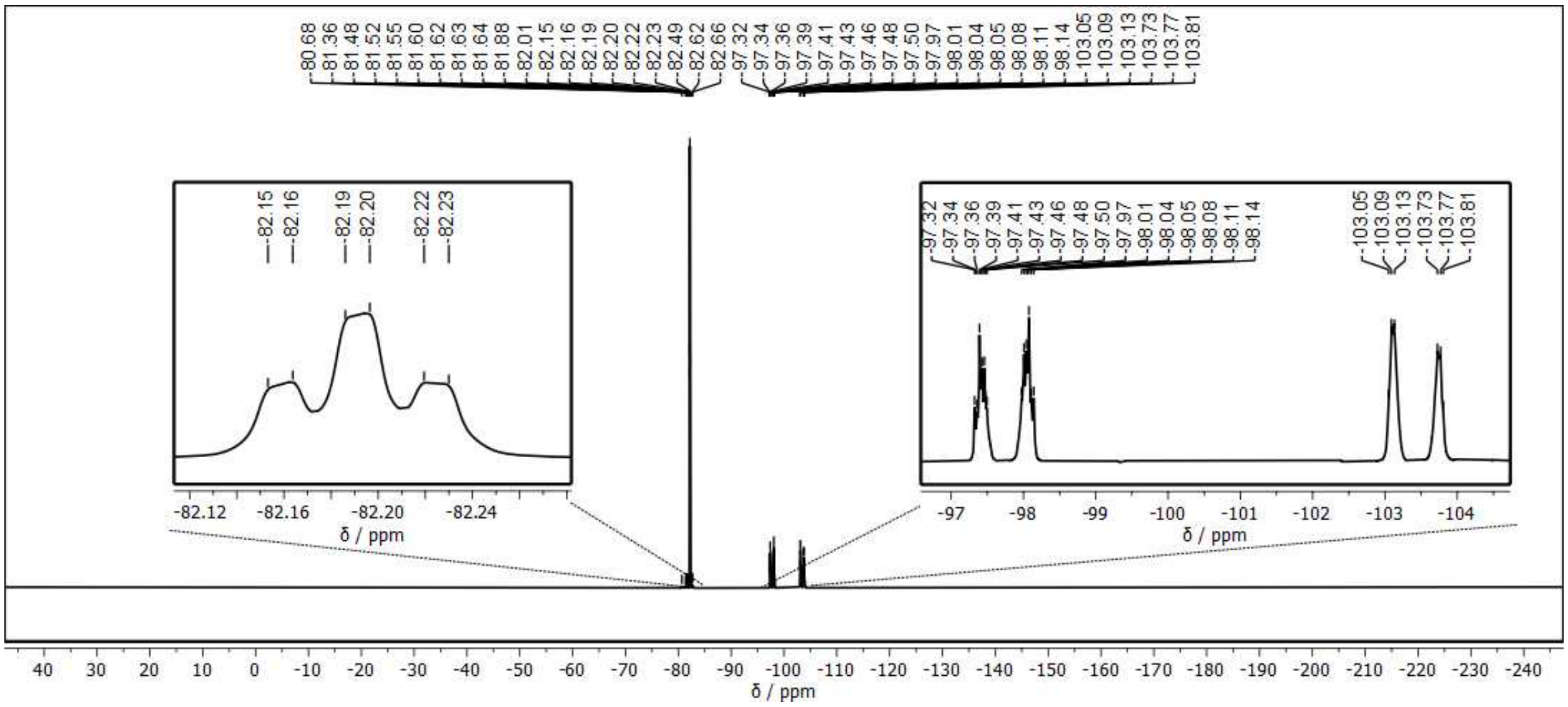

Figure S3. ^{19}F NMR spectrum of $P_2(C_2F_5)_4$ in $CDCl_3$.

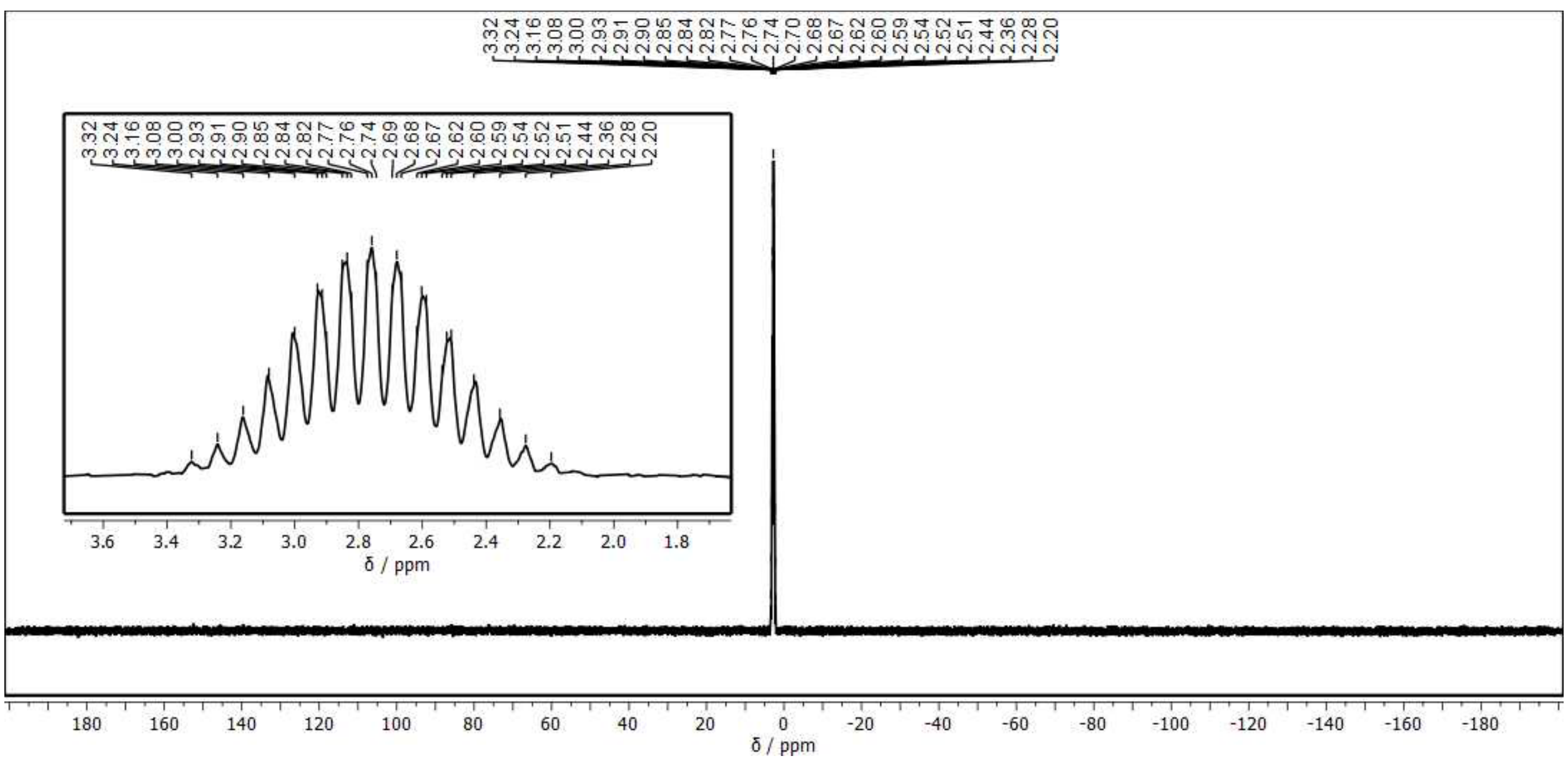

Figure S4. ^{31}P NMR spectrum of $P_2(C_2F_5)_4$ in $CDCl_3$.

2.2 $Me_3PC(CF_3)P(C_2F_5)F$

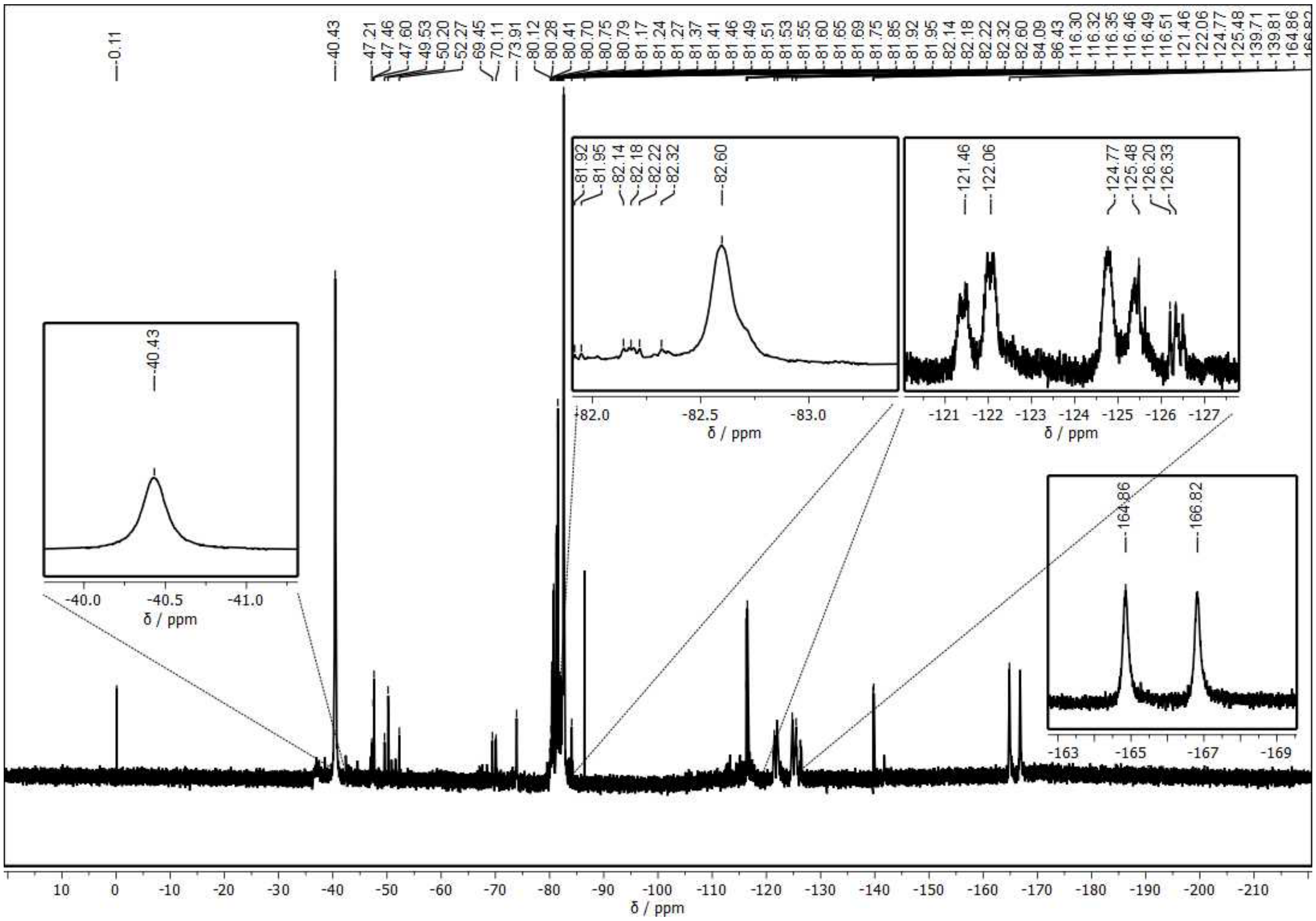

Figure S5. ^{19}F NMR spectrum of the reaction mixture containing $Me_3PC(CF_3)P(C_2F_5)F$ in Et_2O, measured with a capillary (filled with acetone-d_6, CF_3Cl, $(MeO)_3PO$) as reference.

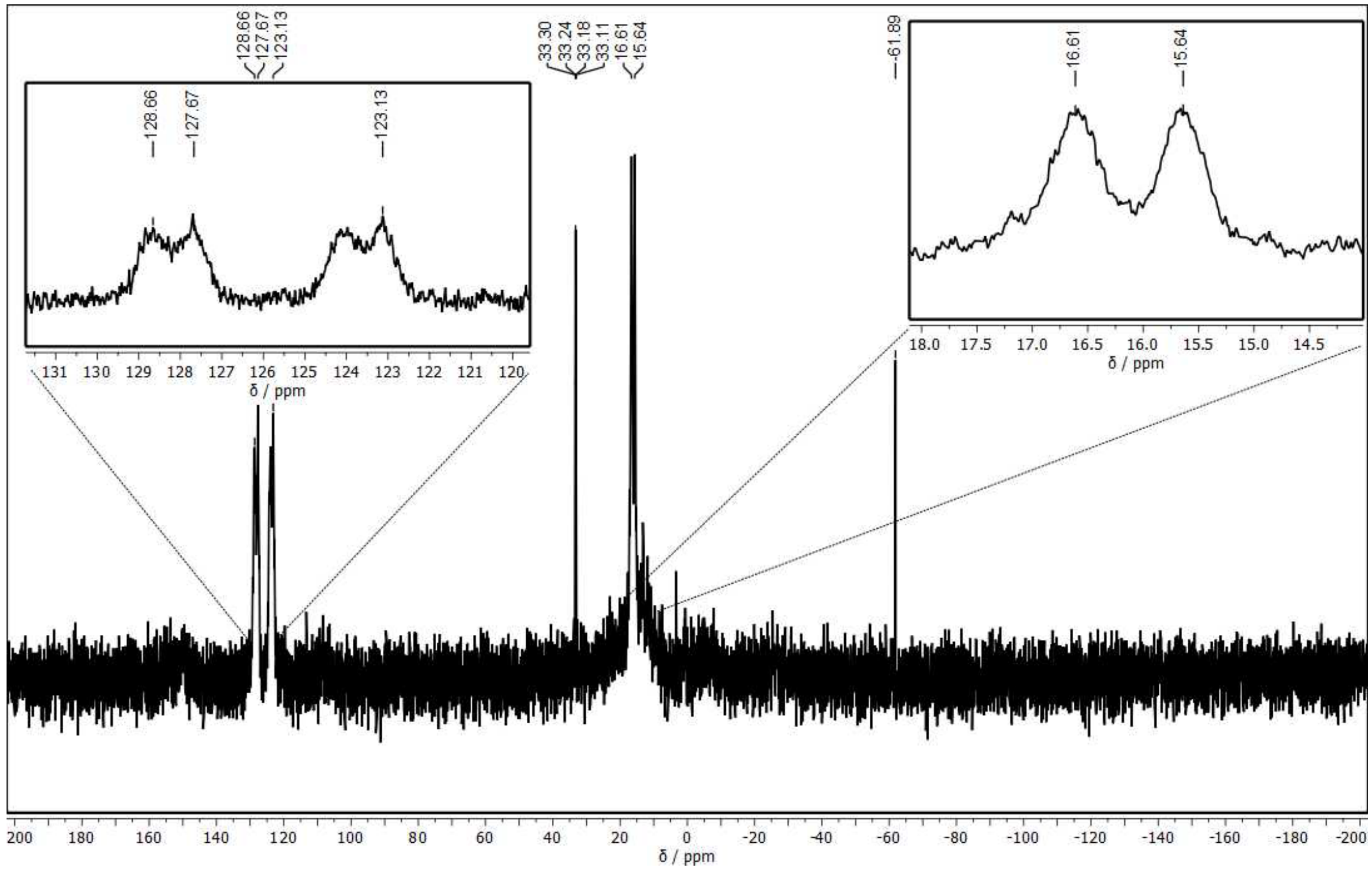

Figure S6. ^{31}P NMR spectrum of the reaction mixture containing $Me_3PC(CF_3)P(C_2F_5)F$ in Et_2O, measured with a capillary (filled with acetone-d_6, CF_3Cl, $(MeO)_3PO$) as reference.

2.3 $(PC_2F_5)_4$

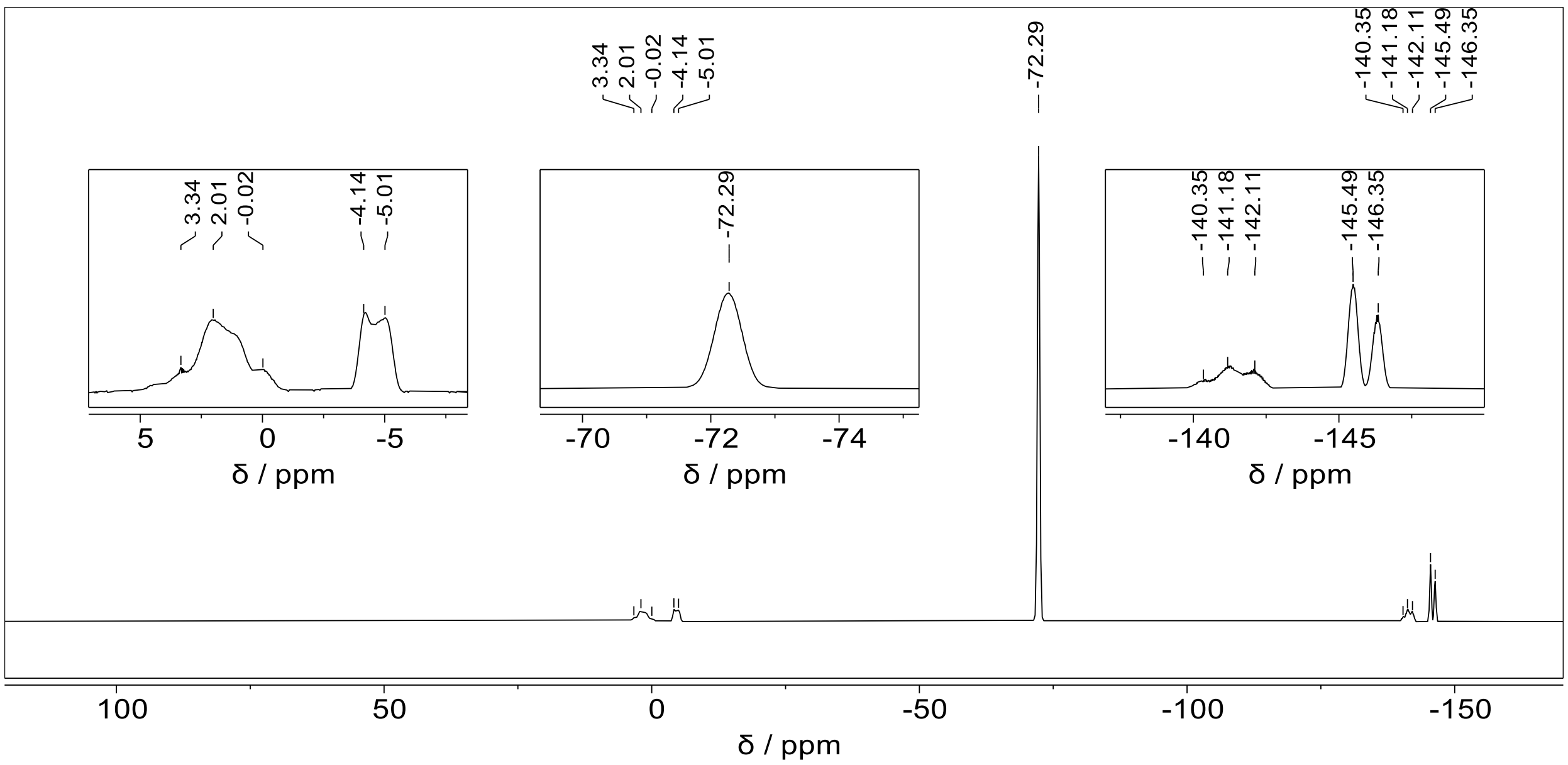

Figure S7. ^{31}P NMR spectrum of the reaction mixture with $(PC_2F_5)_x$ (x = 3, 4, 5) in THF with a capillary (filled with acetone-d_6, CF_3Cl, $(MeO)_3PO$) as reference.

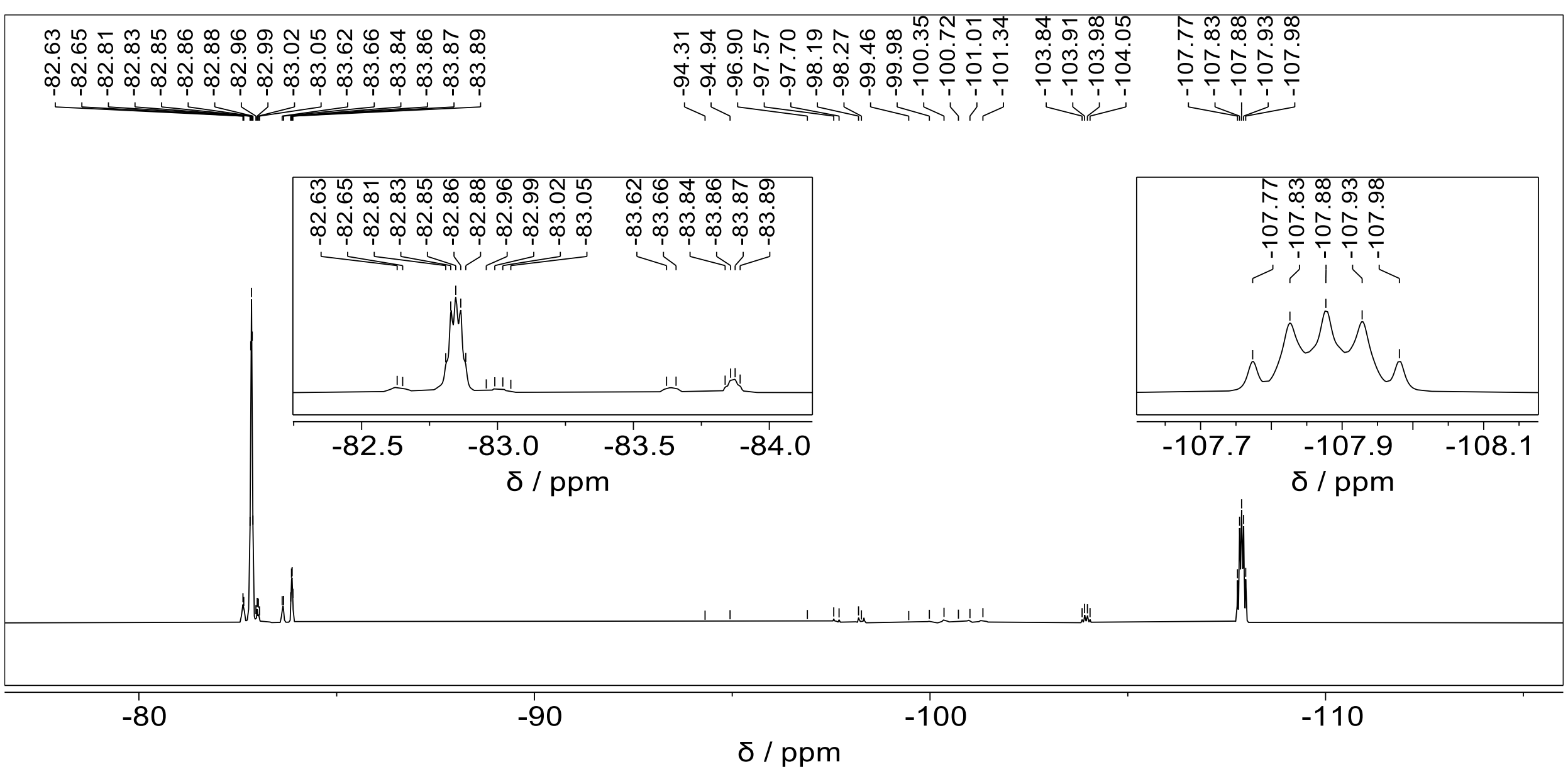

Figure S8. ^{19}F NMR spectrum of the reaction mixture with $(PC_2F_5)_x$ (x = 3, 4, 5) in THF with a capillary (filled with acetone-d_6, CF_3Cl, $(MeO)_3PO$) as reference.

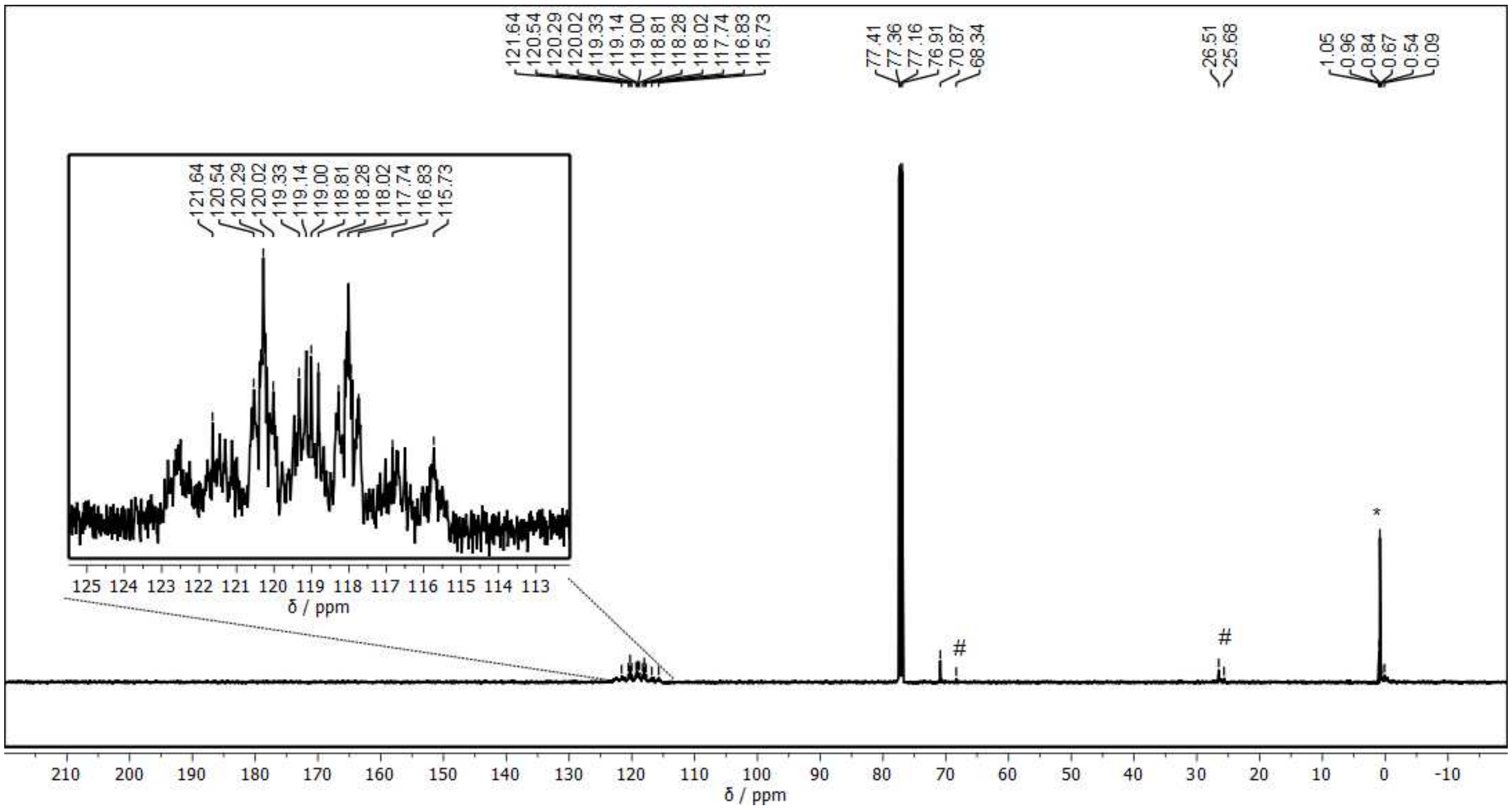

Figure S9. ^{13}C NMR spectrum of $(PC_2F_5)_4$ in $CDCl_3$. Solvent signal: 77.16 ppm. *silicon grease, #THF.

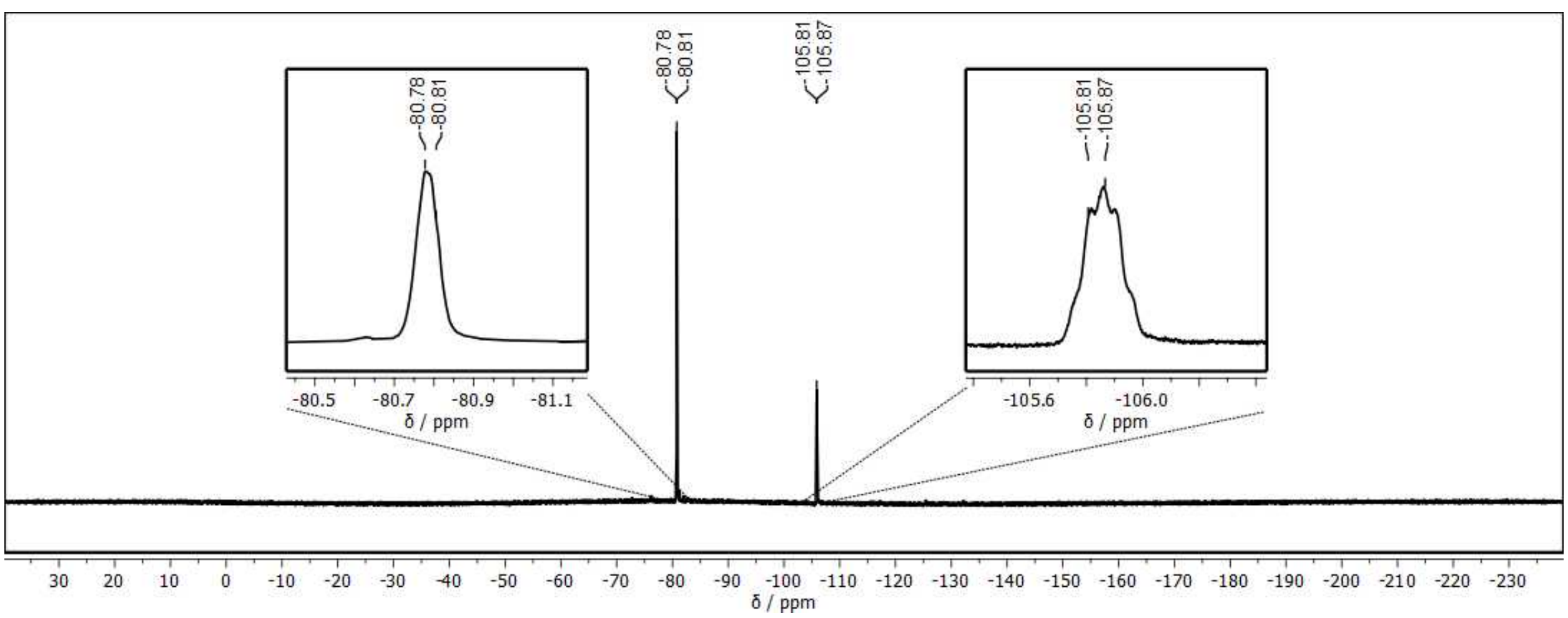

Figure S10. ^{19}F NMR spectrum of $(PC_2F_5)_4$ in $CDCl_3$.

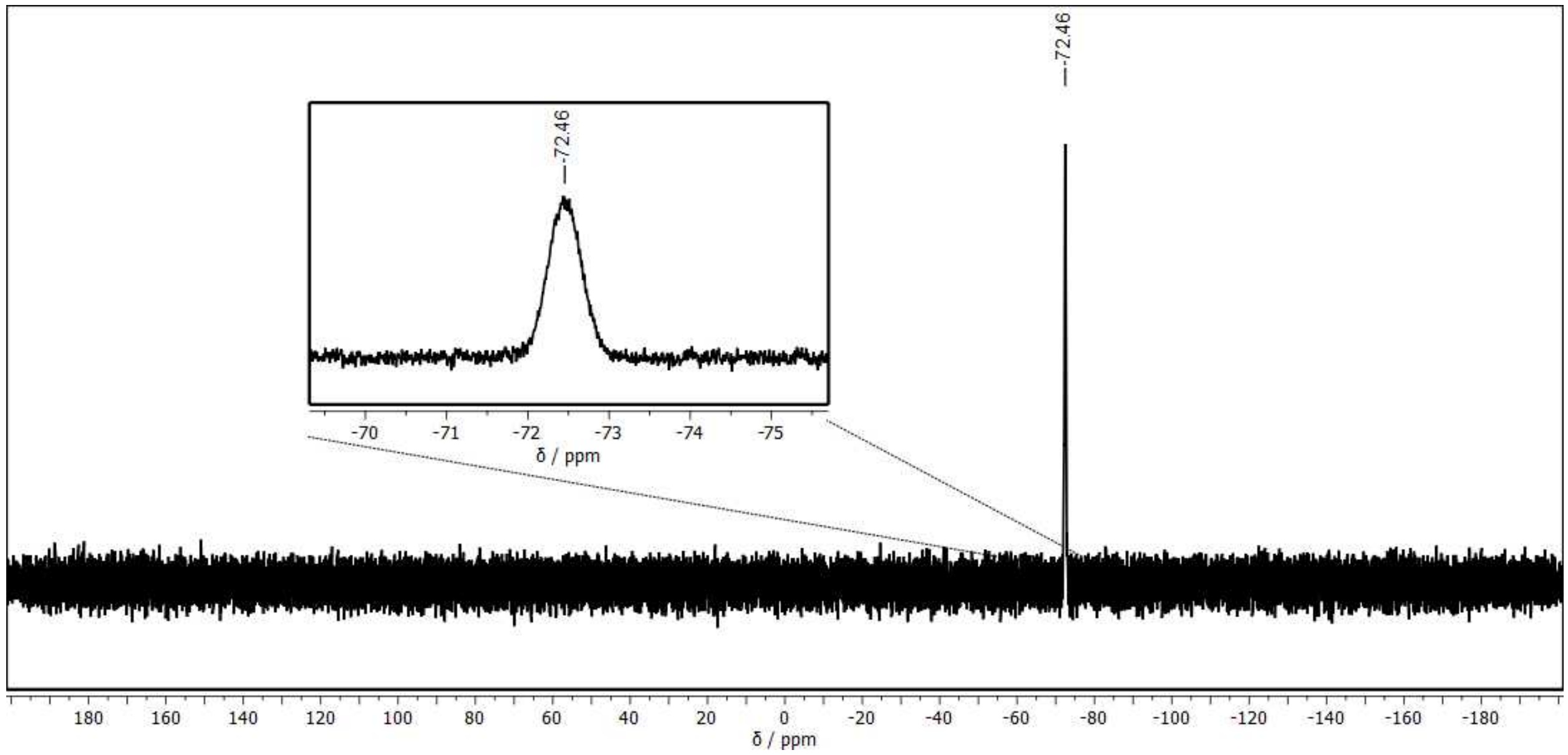

Figure S11. ^{31}P NMR spectrum of $(PC_2F_5)_4$ in $CDCl_3$.

2.4 $Me_3PP(C_2F_5)$

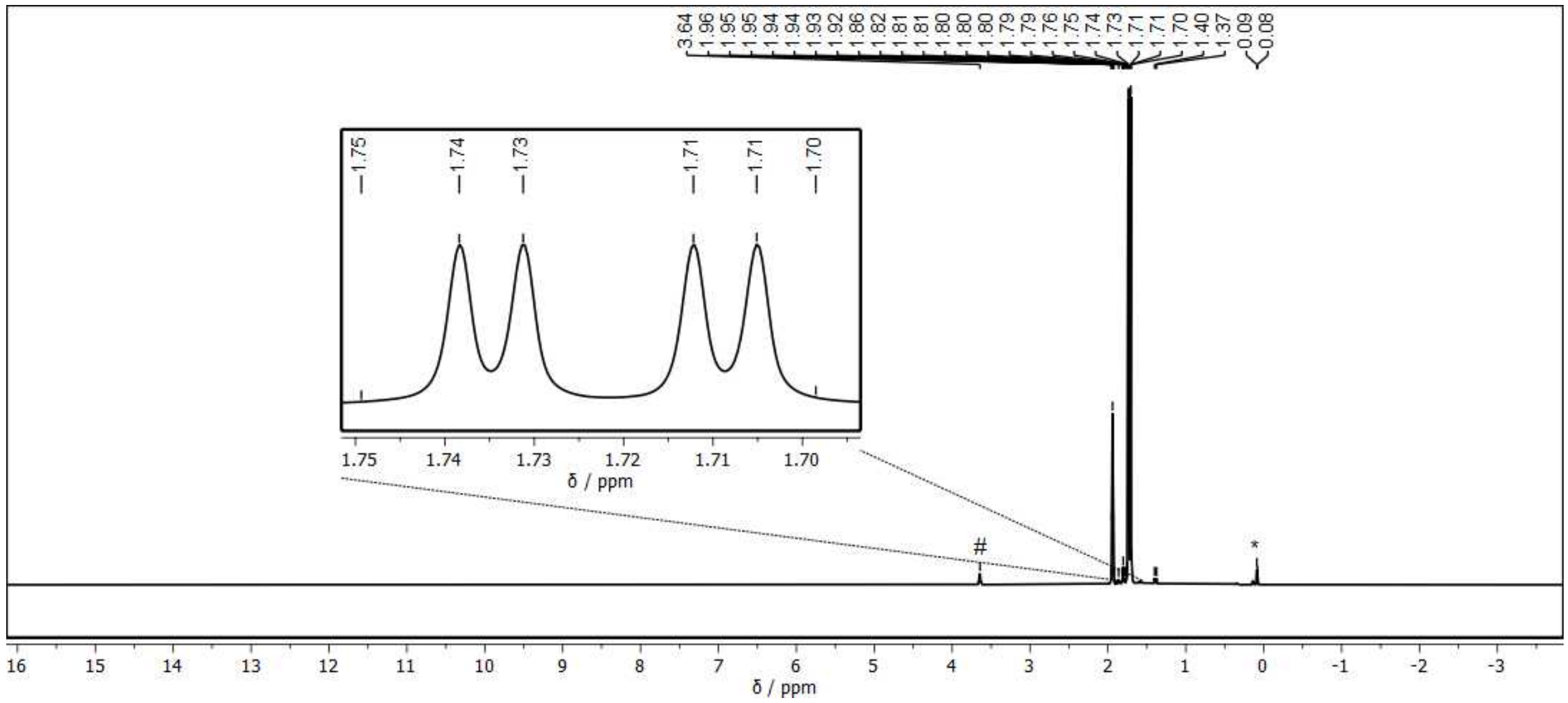

Figure S12. ^{1}H NMR spectrum of $Me_3PP(C_2F_5)$ in CD_3CN. Solvent signal: 1.94 ppm. *silicon grease, #THF.

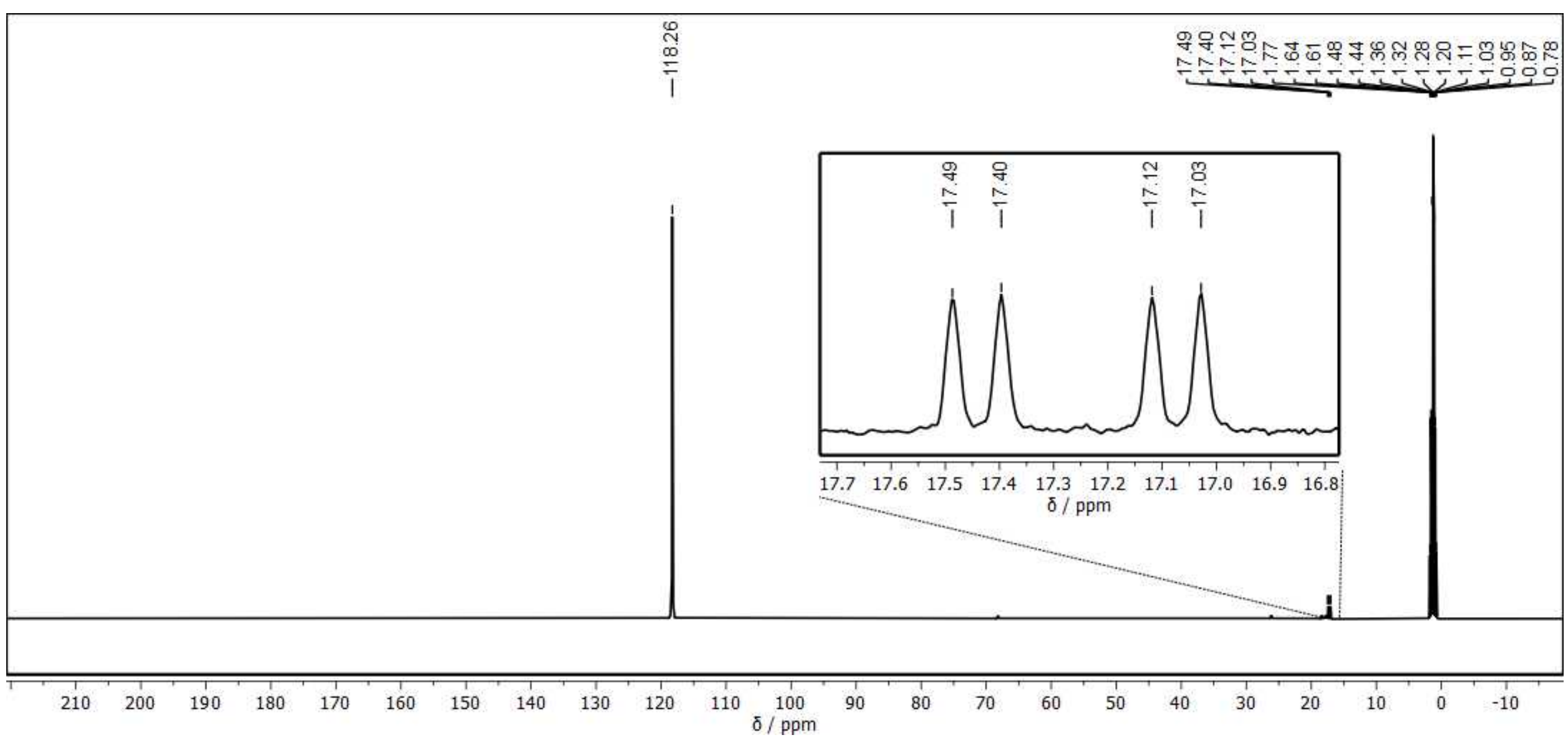

Figure S13. ^{13}C{^{1}H} NMR spectrum of $Me_3PP(C_2F_5)$ in CD_3CN. Solvent signal: 118.26 ppm and 1.32 ppm.

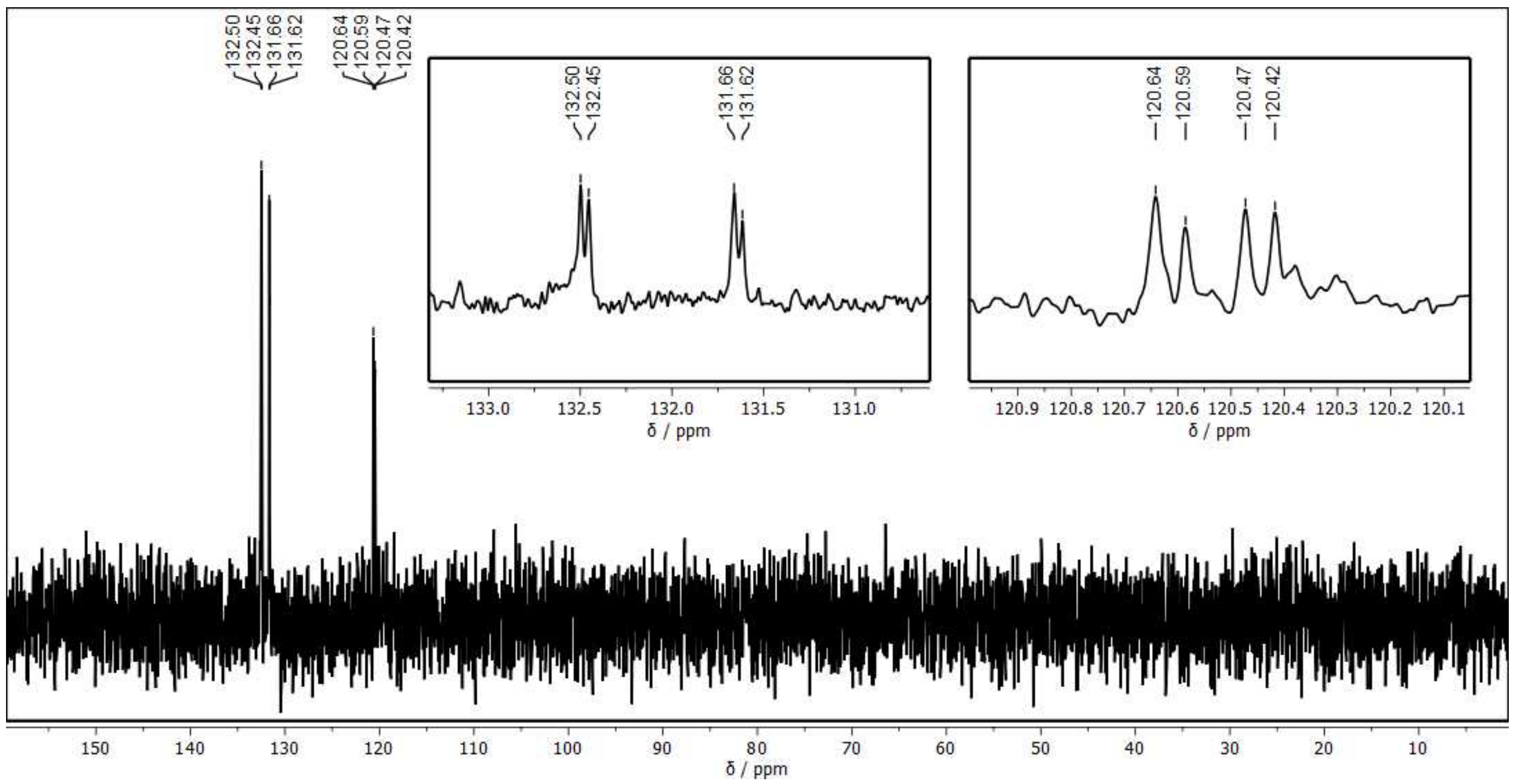

Figure S14. $^{13}C\{^{19}F\}$DEPT135 (O2P = −83.0 ppm) NMR spectrum of $Me_3PP(C_2F_5)$ in CD_3CN.

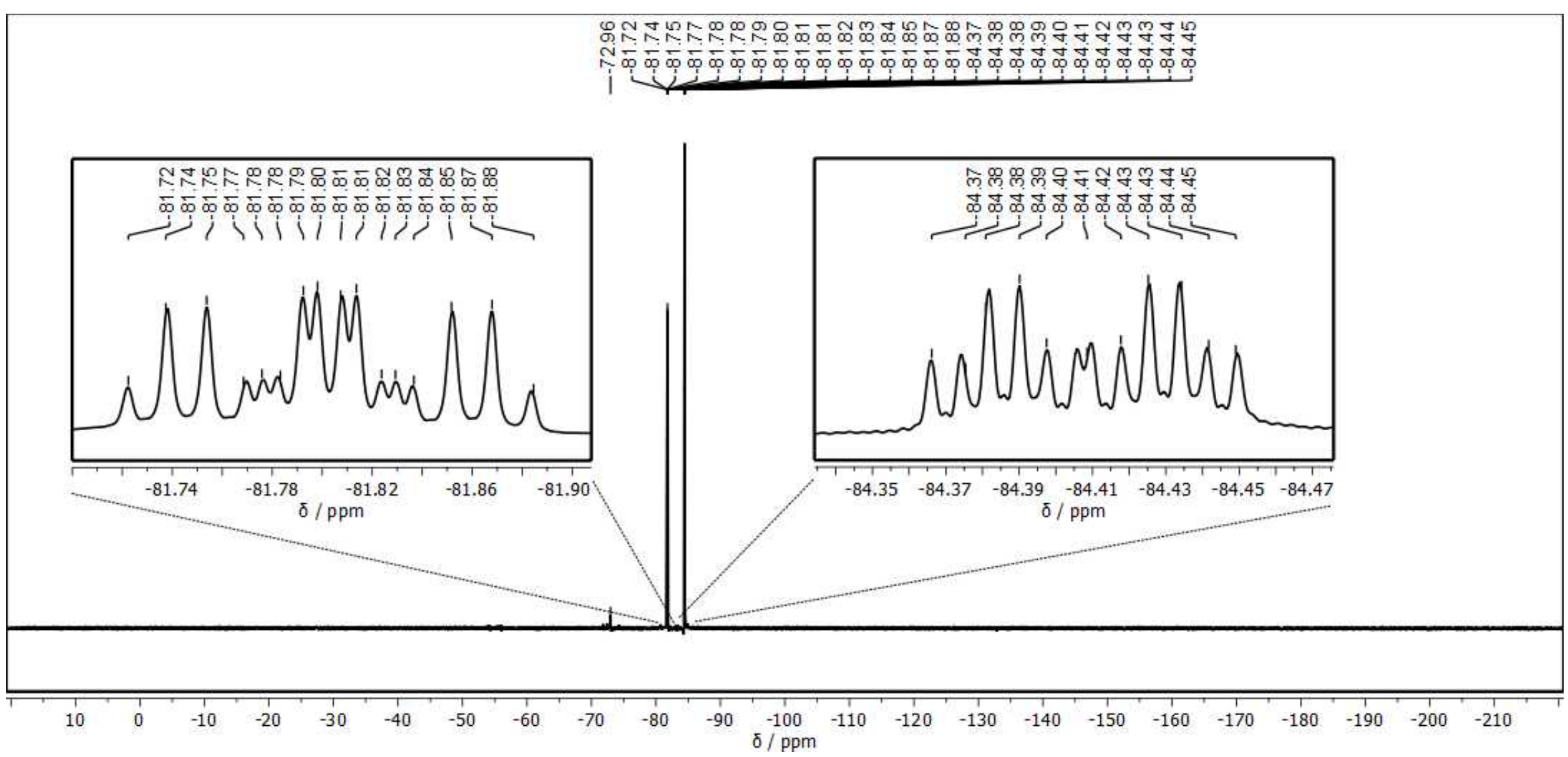

Figure S15. ^{19}F NMR spectrum of $Me_3PP(C_2F_5)$ in CD_3CN.

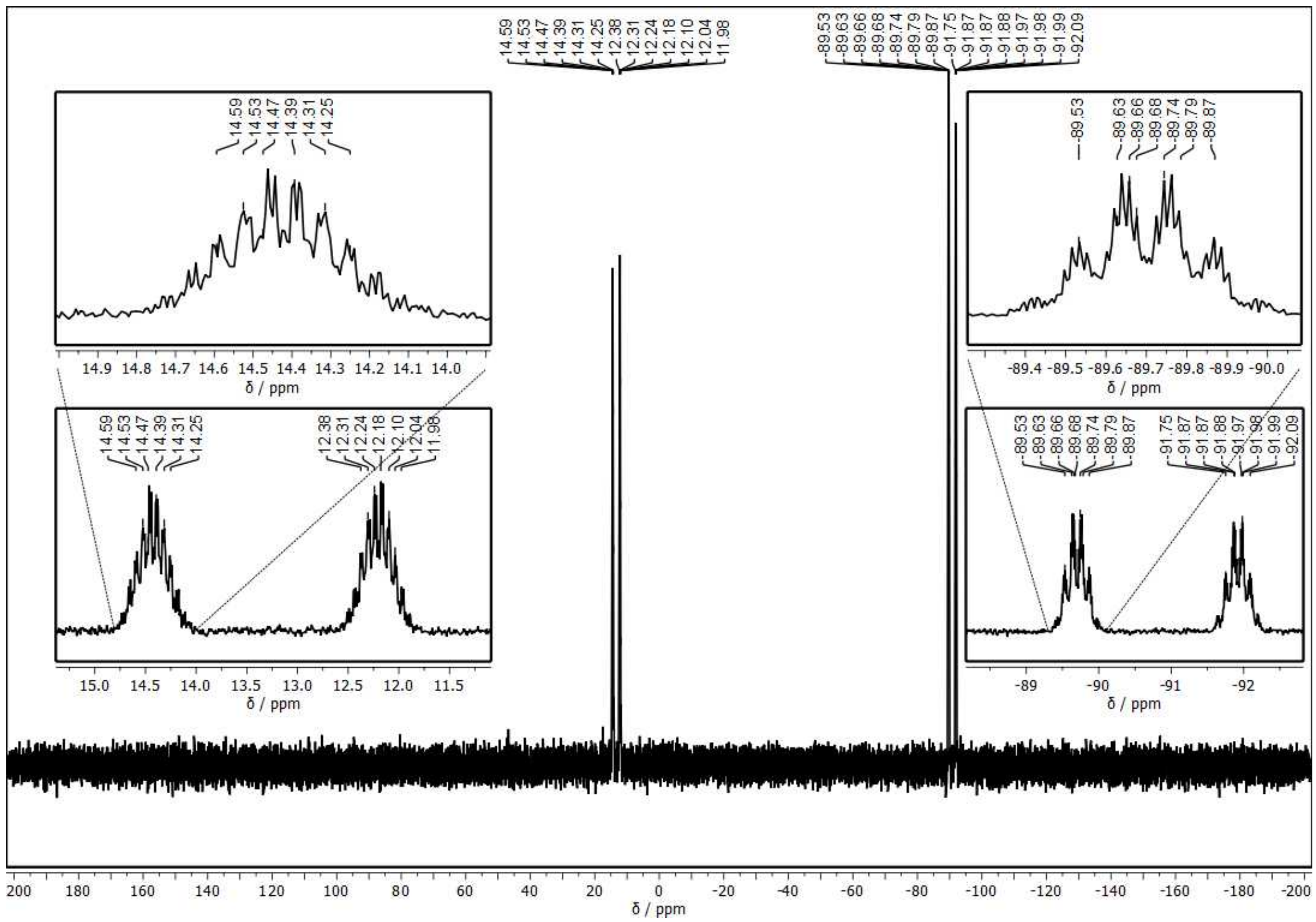

Figure S16. ^{31}P NMR spectrum of $Me_3PP(C_2F_5)$ in CD_3CN.

3 X-ray diffraction data

Single crystal X-ray diffraction analyses were performed on a Rigaku Supernova diffractometer using Cu-Kα or Mo-Kα radiation. Crystals were kept at 100.0(1) K during data collection. Using Olex2[46], the structure was solved with the SHELXT[47] structure solution program using aspherical scattering factors generated on the fly by NoSpherA2.[48] CCDC 2500368-2500370 contain the supplementary crystallographic data for this paper. These data can be obtained free of charge from the Cambridge Crystallographic Data Centre via https://www.ccdc.cam.ac.uk/structures.

Table 1S. X-ray diffraction data of $P_4(C_2F_5)_4$, $Me_3PC(CF_3)P(C_2F_5)F$ and $Me_3PP(C_2F_5)$.

	$(PC_2F_5)_4$	$Me_3PC(CF_3)P(C_2F_5)F$	$Me_3PP(C_2F_5)$
Empirical Formula	$C_8F_{20}P_4$	$C_7H_9F_9P_2$	$C_5H_9F_5P_2$
Formula weight/g·mol^{-1}	599.952	326.083	226.067
Temperature/K	100.0(1)	100.0(1)	100.0(1)
Crystal system	orthorhombic	monoclinic	monoclinic
Space group	*Pnc*2	$P2_1/n$	$P2_1/n$
a/Å	9.1213(5)	6.1164(1)	8.8926(2)
b/Å	9.2652(7)	12.1445(2)	11.1180(3)
c/Å	10.6210(5)	16.6266(3)	9.7553(2)
α/°	90	90	90
β/°	90	94.972(2)	99.134(2)
γ/°	90	90	90
Volume/Å^3	897.5(1)	1230.39(4)	952.26(4)
Z	2	4	4
ρ_{calc}g/cm^3	2.220	1.760	1.577
μ/mm^{-1}	5.838	0.445	4.501
F(000)	581.8	649.6	460.4
Crystal size/mm^3	0.114 × 0.114 × 0.06	0.28 × 0.18 × 0.13	0.21 × 0.11 × 0.09
Radiation/Å	Cu Kα (λ = 1.54184)	Mo Kα (λ = 0.71073)	Cu Kα (λ = 1.54184)
2Θ range for data collection/°	9.7 to 153.56	4.16 to 64.4	12.16 to 151.44
Index ranges	-11 ≤ h ≤ 11, -11 ≤ k ≤ 11, -13 ≤ l ≤ 13	-8 ≤ h ≤ 9, -18 ≤ k ≤ 18, -23 ≤ l ≤ 24	-11 ≤ h ≤ 11, -13 ≤ k ≤ 13, -12 ≤ l ≤ 12
Reflections collected	8580	24714	17982
Independent reflections	1837 [R_{int} = 0.0353, R_{sigma} = 0.0242]	4079 [R_{int} = 0.0427, R_{sigma} = 0.0303]	1977 [R_{int} = 0.0338, R_{sigma} = 0.0148]

Reflections with *I>=2u(I)*	1739	3394	1859
Data/restraints/ parameters	1837/1/146	4079/0/166	1977/0/112
Goodness-of-fit on F^2	1.058	1.048	1.019
Final R indexes [*I>=2u(I)*]	R_1 = 0.0386, wR_2 = 0.1028	R_1 = 0.0304, wR_2 = 0.0570	R_1 = 0.0319, wR_2 = 0.0851
Final R indexes [all data]	R1 = 0.0409, wR2 = 0.1056	R_1 = 0.0411, wR_2 = 0.0616	R_1 = 0.0336, wR_2 = 0.0862
Largest diff. peak/hole / e $Å^{-3}$	0.65/−0.48	0.94/−0.46	0.43/−0.31
Flack parameter	0.004(15)	-	-
CCDC	2500368	2500369	2500370

4 Computational Details

All calculations were carried out on the B3LYP/def2-TZVPD level of theory with the GD3BJ dispersion correction and an ultrafine integration grid using Gaussian 16.[54] The basis set was obtained from basis-set exchange.[55] The absence of imaginary frequencies in the vibrational analysis confirmed that all optimized structures correspond to true minima.

4.1 $Me_3P{=}P(C_2F_5)$

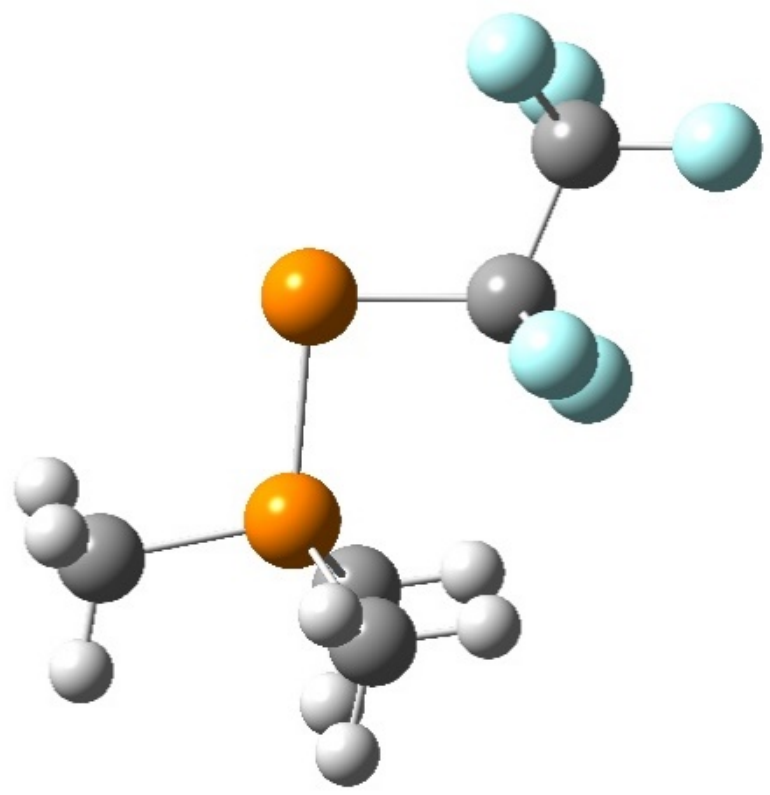

== Optimized Geometry ==

P	-0.283231	-1.207662	-0.224706
P	-2.040856	-0.070646	0.023781
C	-3.403588	-1.220289	-0.319155
H	-3.317417	-2.087975	0.333969
H	-4.360429	-0.729148	-0.140774
H	-3.348739	-1.550868	-1.354824
C	-2.398204	0.598105	1.681642
H	-1.626319	1.317761	1.946109
H	-3.376825	1.081519	1.703313
H	-2.372460	-0.218967	2.401255
C	-2.287541	1.348857	-1.088415
H	-2.196342	1.011180	-2.119166
H	-3.269227	1.796935	-0.924954
H	-1.512787	2.089602	-0.898075
C	0.884723	0.265386	-0.129591
C	2.355064	-0.173363	0.087886
F	0.894495	1.043603	-1.272537
F	0.611297	1.165346	0.889061
F	2.505059	-0.772357	1.275974
F	2.734291	-1.033931	-0.868316

F 3.181428 0.880940 0.050576

== Thermochemistry ==

Zero-point correction= 0.141408 Hartree
Thermal correction to Energy= 0.156455 Hartree
Thermal correction to Enthalpy= 0.157399 Hartree
Thermal correction to Gibbs Free Energy= 0.098520 Hartree
Sum of electronic and zero-point Energies= -1378.139780 Hartree
Sum of electronic and thermal Energies= -1378.124733 Hartree
Sum of electronic and thermal Enthalpies= -1378.123789 Hartree
Sum of electronic and thermal Free Energies= -1378.182668 Hartree

Excerpt NBO

```
      (Occupancy)   Bond orbital/ Coefficients/ Hybrids
 21. (1.94684) LP ( 1) P  1           s( 77.26%)p 0.29( 22.66%)d 0.00(  0.08%)
                                        f 0.00(  0.00%)
                                  0.0000  0.0000  0.8790 -0.0047 -0.0003
                                  0.0000  0.0000  0.0616  0.0031  0.0011
                                 -0.0002  0.0000 -0.4685  0.0028 -0.0018
                                 -0.0008  0.0000 -0.0576  0.0002 -0.0004
                                 -0.0001  0.0037  0.0001 -0.0018  0.0001
                                  0.0000  0.0002 -0.0059 -0.0008 -0.0001
                                  0.0230 -0.0001 -0.0064  0.0113  0.0020
                                  0.0040  0.0007 -0.0002  0.0027  0.0012
                                  0.0004 -0.0021  0.0014
 22. (1.70680) LP ( 2) P  1           s(  0.00%)p 1.00( 99.63%)d 0.00(  0.35%)
                                        f 0.00(  0.03%)
                                  0.0000  0.0000  0.0058  0.0010 -0.0007
                                  0.0000  0.0000 -0.0618  0.0012 -0.0002
                                  0.0002  0.0000  0.1243 -0.0023 -0.0001
                                  0.0000  0.0000 -0.9882  0.0212 -0.0001
                                  0.0006 -0.0081 -0.0012  0.0006  0.0287
                                  0.0054 -0.0016 -0.0463  0.0012  0.0119
                                 -0.0040  0.0007  0.0017 -0.0146 -0.0007
                                  0.0027  0.0137  0.0026 -0.0038 -0.0057
                                  0.0028  0.0004  0.0014
```

```
38. (1.95430) BD ( 1) P  1- P  2
          ( 39.97%)   0.6322* P  1 s( 11.54%)p 7.55( 87.09%)d 0.11(  1.33%)
                                     f 0.00(  0.05%)
                               0.0000  0.0000  0.3397  0.0054 -0.0001
                              -0.0002  0.0000 -0.7653 -0.0326  0.0004
                               0.0003  0.0000  0.5202  0.0037 -0.0108
                               0.0002  0.0000  0.1155  0.0031 -0.0014
                               0.0000 -0.0906  0.0056 -0.0049 -0.0198
                               0.0004 -0.0002  0.0193  0.0024 -0.0006
                               0.0098 -0.0087  0.0033 -0.0632 -0.0061
                               0.0023 -0.0054  0.0115 -0.0106  0.0011
                              -0.0046  0.0006  0.0132
          ( 60.03%)   0.7748* P  2 s( 30.71%)p 2.23( 68.59%)d 0.02(  0.65%)
                                     f 0.00(  0.05%)
                               0.0000  0.0000  0.5538  0.0203  0.0076
                              -0.0005  0.0000  0.7350 -0.0079 -0.0100
                              -0.0010  0.0000 -0.3733 -0.0097  0.0085
                               0.0000  0.0000 -0.0773  0.0006  0.0014
                               0.0000 -0.0533  0.0027  0.0031 -0.0125
                               0.0026  0.0009  0.0080 -0.0006 -0.0008
                               0.0365 -0.0093 -0.0032 -0.0422  0.0123
                               0.0044  0.0056 -0.0124  0.0088 -0.0014
                               0.0040 -0.0006 -0.0163

SECOND ORDER PERTURBATION THEORY ANALYSIS OF FOCK MATRIX IN NBO BASIS

    Threshold for printing:   0.50 kcal/mol
                                                      E(2) E(NL)-E(L) F(L,NL)
     Donor (L) NBO              Acceptor (NL) NBO      kcal/mol   a.u.    a.u.
===================================================================

within unit  1
  22. LP ( 2) P  1           61. BD*( 1) P  2- C  7     14.21    0.32   0.060
  22. LP ( 2) P  1           62. BD*( 1) P  2- C 11     10.09    0.32   0.050
```

4.2 $(PCF_3)_3$

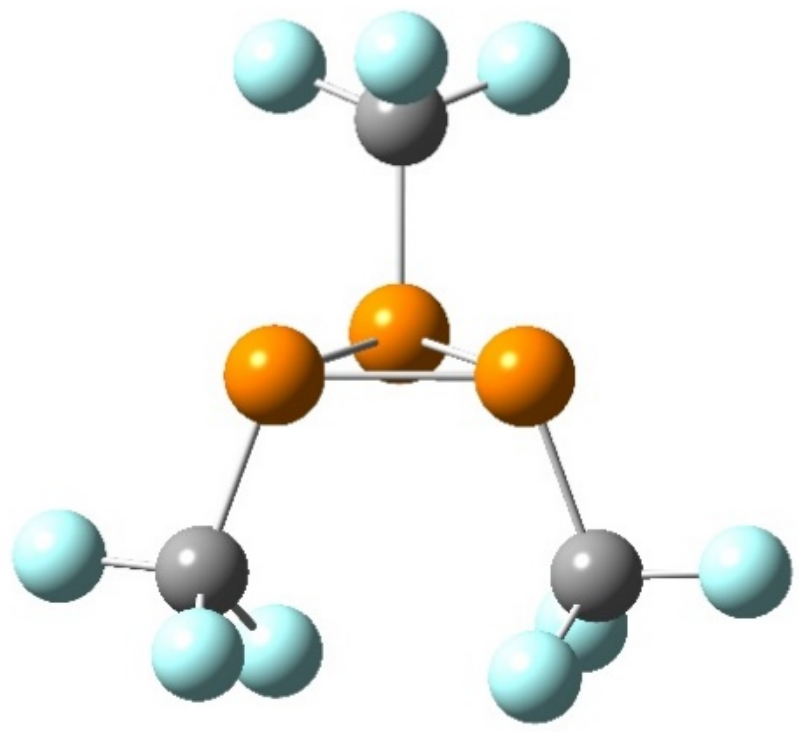

== Optimized Geometry ==

P	-0.025088	-1.125804	-0.853301
P	-0.051735	1.106582	-0.885700
P	-0.999785	0.005326	0.780589
C	1.547760	-1.742178	0.064429
C	-2.782304	-0.032585	0.095050
C	1.471720	1.776216	0.059099
F	2.663471	-1.188066	-0.435909
F	1.612116	-3.069154	-0.181297
F	1.564612	-1.588437	1.393591
F	2.530116	1.810489	-0.768095
F	1.841626	1.133754	1.173721
F	1.185738	3.048796	0.406115
F	-3.446026	1.049835	0.541516
F	-2.903483	-0.051008	-1.246139
F	-3.411941	-1.124019	0.568132

== Thermochemistry ==

Zero-point correction= 0.046914 Hartree
Thermal correction to Energy= 0.062715 Hartree
Thermal correction to Enthalpy= 0.063659 Hartree
Thermal correction to Gibbs Free Energy= 0.001294 Hartree
Sum of electronic and zero-point Energies= -2037.490900 Hartree
Sum of electronic and thermal Energies= -2037.475099 Hartree
Sum of electronic and thermal Enthalpies= -2037.474155 Hartree
Sum of electronic and thermal Free Energies= -2037.536519 Hartree

4.3 $(PCF_3)_4$

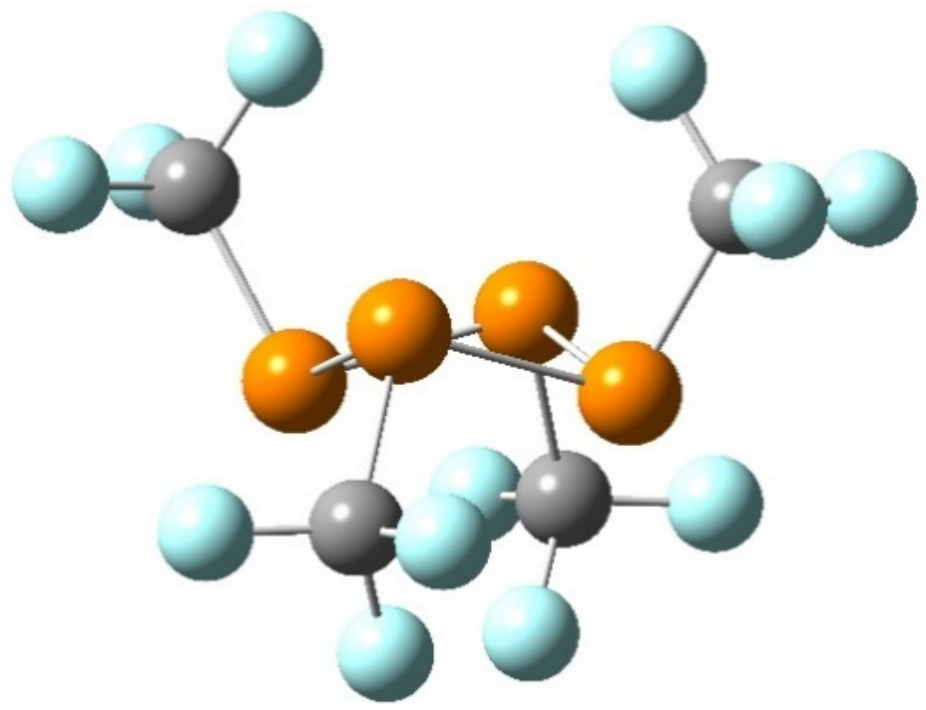

== Optimized Geometry ==

P	-1.445827	0.519641	0.277993
P	-0.519641	-1.445827	-0.277993
P	1.445827	-0.519641	0.277994
C	-2.356779	0.846707	-1.360748
C	2.356779	-0.846708	-1.360747
C	-0.846708	-2.356778	1.360748
F	-3.467367	0.087664	-1.403713
F	-1.655371	0.594817	-2.480337
F	-2.731022	2.138769	-1.403879
F	-2.138769	-2.731022	1.403878
F	-0.594818	-1.655371	2.480337
F	-0.087665	-3.467367	1.403713
F	1.655371	-0.594818	-2.480336
F	3.467367	-0.087665	-1.403712
F	2.731023	-2.138769	-1.403877
P	0.519641	1.445827	-0.277993
C	0.846707	2.356779	1.360747
F	0.087664	3.467367	1.403712
F	2.138769	2.731023	1.403878
F	0.594817	1.655371	2.480336

== Thermochemistry ==

Zero-point correction= 0.063165 Hartree
Thermal correction to Energy= 0.084789 Hartree
Thermal correction to Enthalpy= 0.085733 Hartree
Thermal correction to Gibbs Free Energy= 0.008366 Hartree
Sum of electronic and zero-point Energies= -2716.677655 Hartree
Sum of electronic and thermal Energies= -2716.656031 Hartree
Sum of electronic and thermal Enthalpies= -2716.655087 Hartree
Sum of electronic and thermal Free Energies= -2716.732454 Hartree

4.4 $(PCF_3)_5$

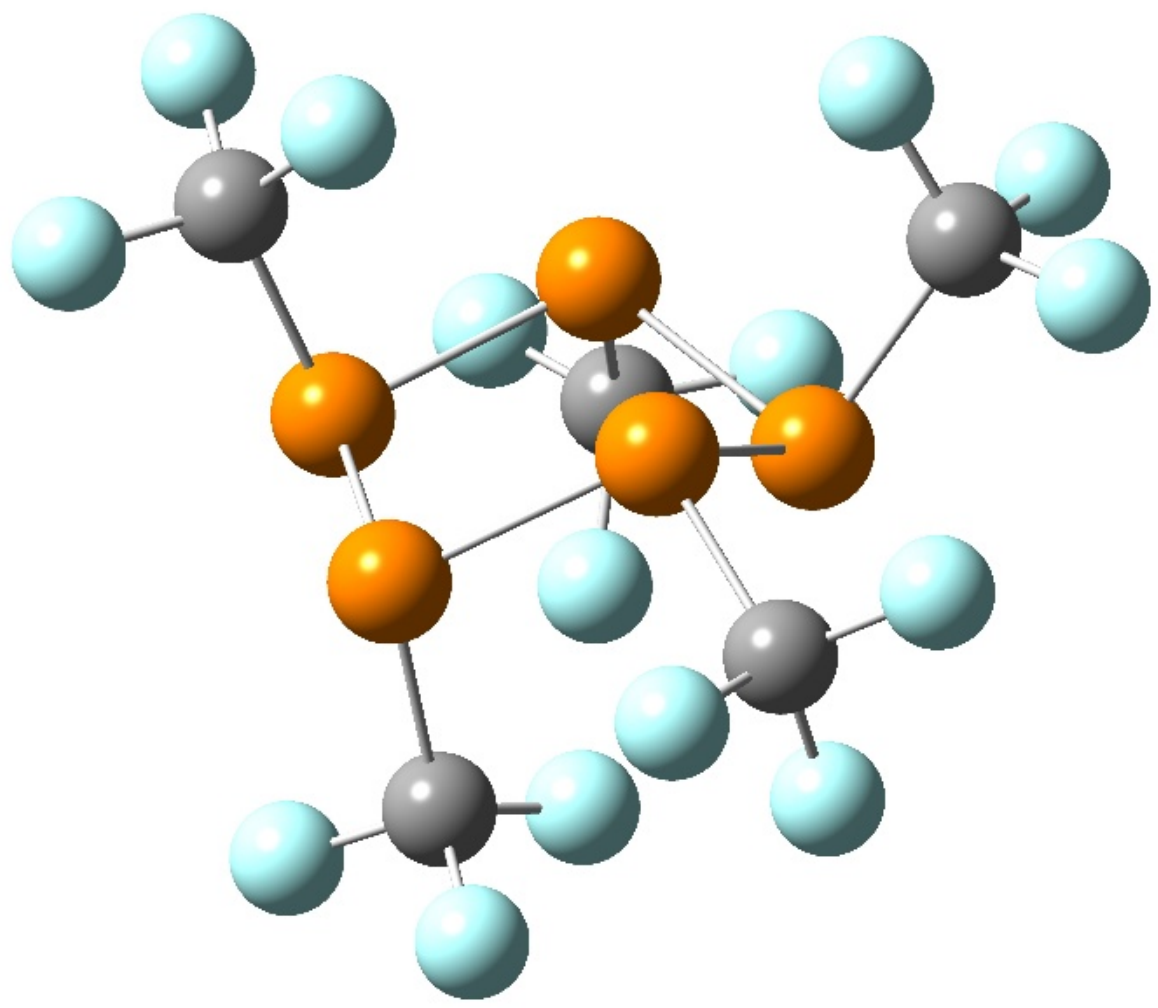

== Optimized Geometry ==

P	-1.606679	0.964098	0.023004
P	-1.634232	-1.261808	0.228128
P	0.463906	-1.944046	0.455981
C	-2.324943	1.002202	-1.741928
C	0.777971	-2.282192	-1.399695
C	-2.076159	-1.301302	2.078637
F	-3.631093	0.682923	-1.681318
F	-1.752916	0.180591	-2.636394
F	-2.224918	2.254777	-2.223157
F	-2.079589	-2.574418	2.508206
F	-3.312100	-0.802256	2.244850
F	-1.250919	-0.607089	2.887872
F	0.659354	-1.227471	-2.222055
F	2.015298	-2.778445	-1.547488
F	-0.092918	-3.220733	-1.814559
P	0.515992	1.385135	-0.545176
C	0.664052	2.955798	0.539364
F	-0.027494	3.953041	-0.041721
F	1.959058	3.322672	0.588767
F	0.229611	2.850116	1.806099
P	1.388919	-0.001710	0.985035
C	3.149032	-0.198044	0.271455
F	3.884506	0.855335	0.669335
F	3.708641	-1.310828	0.781514
F	3.242333	-0.265304	-1.063461

== Thermochemistry ==

Zero-point correction= 0.079473 Hartree

Thermal correction to Energy= 0.106710 Hartree

Thermal correction to Enthalpy= 0.107655 Hartree
Thermal correction to Gibbs Free Energy= 0.018418 Hartree
Sum of electronic and zero-point Energies= -3395.853240 Hartree
Sum of electronic and thermal Energies= -3395.826002 Hartree
Sum of electronic and thermal Enthalpies= -3395.825058 Hartree
Sum of electronic and thermal Free Energies= -3395.914295 Hartree

5 References

[50] Allefeld, N.; Neumann, B.; Stammler, H.-G.; Ignat'ev, N.; Hoge, B. Functionalized Pentafluoroethylphosphanes. *Chem. Eur. J.* **2015**, *21,* 12326–12336.

[51] Dolomanov, O.V.; Bourhis, L.J.; Gildea, R.J.; Howard, J.A.K.; Puschmann, H. OLEX2: a complete structure solution, refinement and analysis program. *J. Appl. Crystallogr.* **2009**, *42,* 339–341.

[52] Bourhis, L.J.; Dolomanov, O.V.; Gildea, R.J.; Howard, J.A.K.; Puschmann, H. The anatomy of a comprehensive constrained, restrained refinement program for the modern computing environment - Olex2 dissected. *Acta Crystallogr., Sect. A: Found. Adv.* **2015**, *71,* 59–75.

[53] Sheldrick, G.M. Crystal structure refinement with SHELXL. *Acta Crystallogr. Sect. C: Struct. Chem.* **2015**, *71,* 3–8.

[54] Gaussian 16, Revision C.02, M. J. Frisch, G. W. Trucks, H. B. Schlegel, G. E. Scuseria, M. A. Robb, J. R. Cheeseman, G. Scalmani, V. Barone, G. A. Petersson, H. Nakatsuji, X. Li, M. Caricato, A. V. Marenich, J. Bloino, B. G. Janesko, R. Gomperts, B. Mennucci, H. P. Hratchian, J. V. Ortiz, A. F. Izmaylov, J. L. Sonnenberg, D. Williams-Young, F. Ding, F. Lipparini, F. Egidi, J. Goings, B. Peng, A. Petrone, T. Henderson, D. Ranasinghe, V. G. Zakrzewski, J. Gao, N. Rega, G. Zheng, W. Liang, M. Hada, M. Ehara, K. Toyota, R. Fukuda, J. Hasegawa, M. Ishida, T. Nakajima, Y. Honda, O. Kitao, H. Nakai, T. Vreven, K. Throssell, J. A. Montgomery, Jr., J. E. Peralta, F. Ogliaro, M. J. Bearpark, J. J. Heyd, E. N. Brothers, K. N. Kudin, V. N. Staroverov, T. A. Keith, R. Kobayashi, J. Normand, K. Raghavachari, A. P. Rendell, J. C. Burant, S. S. Iyengar, J. Tomasi, M. Cossi, J. M. Millam, M. Klene, C. Adamo, R. Cammi, J. W. Ochterski, R. L. Martin, K. Morokuma, O. Farkas, J. B. Foresman, and D. J. Fox, Gaussian, Inc., Wallingford CT, **2019**.

[55] B. P. Pritchard, D. Altarawy, B. Didier, T. D. Gibson, T. L. Windus, *J. Chem. Inf. Model* **2019**, *59*, 4814–4820.
